# Evaluation of Phage Display Biopanning Strategies for the Selection of Anti-Cell Surface Receptor Antibodies

**DOI:** 10.3390/ijms23158470

**Published:** 2022-07-30

**Authors:** Nadya Panagides, Lucia F. Zacchi, Mitchell J. De Souza, Rodrigo A. V. Morales, Alexander Karnowski, Mark T. Liddament, Catherine M. Owczarek, Stephen M. Mahler, Con Panousis, Martina L. Jones, Christian Fercher

**Affiliations:** 1 ARC Training Centre for Biopharmaceutical Innovation, Australian Institute for Bioengineering and Nanotechnology (AIBN), University of Queensland, Brisbane, QLD 4072, Australia; n.panagides@uq.edu.au (N.P.); l.zacchi@uq.edu.au (L.F.Z.); s.mahler@eng.uq.edu.au (S.M.M.); martina.jones@uq.edu.au (M.L.J.); 2 Research and Development, CSL Limited, Bio21 Molecular Science and Biotechnology Institute, Parkville, VIC 3052, Australia; mitchell.desouza@csl.com.au (M.J.D.S.); rodrigo.morales@csl.com.au (R.A.V.M.); alexander.karnowski@csl.com.au (A.K.); mark.liddament@csl.com.au (M.T.L.); catherine.owczarek@csl.com.au (C.M.O.); kosta.panousis@csl.com.au (C.P.)

**Keywords:** biopanning, phage display, mouse common beta receptor, monoclonal antibodies, functional inhibition

## Abstract

Monoclonal antibodies (mAbs) are one of the most successful and versatile protein-based pharmaceutical products used to treat multiple pathological conditions. The remarkable specificity of mAbs and their affinity for biological targets has led to the implementation of mAbs in the therapeutic regime of oncogenic, chronic inflammatory, cardiovascular, and infectious diseases. Thus, the discovery of novel mAbs with defined functional activities is of crucial importance to expand our ability to address current and future clinical challenges. In vitro, antigen-driven affinity selection employing phage display biopanning is a commonly used technique to isolate mAbs. The success of biopanning is dependent on the quality and the presentation format of the antigen, which is critical when isolating mAbs against membrane protein targets. Here, we provide a comprehensive investigation of two established panning strategies, surface-tethering of a recombinant extracellular domain and cell-based biopanning, to examine the impact of antigen presentation on selection outcomes with regards to the isolation of positive mAbs with functional potential against a proof-of-concept type I cell surface receptor. Based on the higher sequence diversity of the resulting antibody repertoire, presentation of a type I membrane protein in soluble form was more advantageous over presentation in cell-based format. Our results will contribute to inform and guide future antibody discovery campaigns against cell surface proteins.

## 1. Introduction

Since first described in 1990, in vitro selection of specific antibody fragments by phage display has revolutionized the field of monoclonal antibody (mAb) discovery [1]. The isolation of phages that bind specifically to the target antigen from a library of millions of sequences derived from either native or synthetic immune repertoires has substantially expanded our ability to generate novel mAbs beyond the immunization of animals. Biopanning is an affinity selection technique used to identify phage display variants with desired binding properties towards a given target [2]. To date, 14 mAbs derived from phage display have been approved for clinical use for the treatment of a variety of diseases, including rheumatoid arthritis, anthrax, macular degeneration, and several malignancies such as gastric, colorectal, and non-small cell lung carcinoma [3]. Moreover, there are currently dozens of antibodies in pre-clinical development and in various phases of clinical testing that have been identified by phage display technology [3].

The identification of target-specific mAbs requires the exposure of a vast collection of antibody variants to the target antigen. To isolate antibodies capable of binding the native target, the protein must be presented in a format that recapitulates its native structure. However, the inherent hydrophobic nature of many transmembrane proteins poses a challenge for antigen presentation [4]. Hydrophobic proteins have been employed for biopanning either as truncated soluble recombinant extracellular domains (ECDs) or reconstituted in an artificial lipid environment such as liposomes [5] or nanodiscs [6]. Other approaches to present antigens from hydrophobic regions include the introduction of stabilizing mutations typically focused on transmembrane helices [7], the use of purified target-enriched cell membranes [8], and the use of scaffold proteins to present grafted native epitopes [9]. Recombinant ECDs may not be ideal for the isolation of target-specific antibodies due to the potential for non-native folding and the removal or inclusion of epitopes resulting from the truncation process or epitopes previously inaccessible in the membrane-bound state. Moreover, many membrane proteins do not have large enough ECDs that fold independently when expressed recombinantly or are unstable when isolated from their native context [10]. Conversely, cell-based presentation methods that preserve the native protein structure may be hampered by the low-level expression of the target antigen, and there may be interference by the endogenous cell surface proteome. Thus, both surface-tethered recombinant extracellular domain (STRE) and cell-based antigen (CBA) presentation styles present advantages and challenges that need to be explored in a target-based approach.

**Figure 1:**
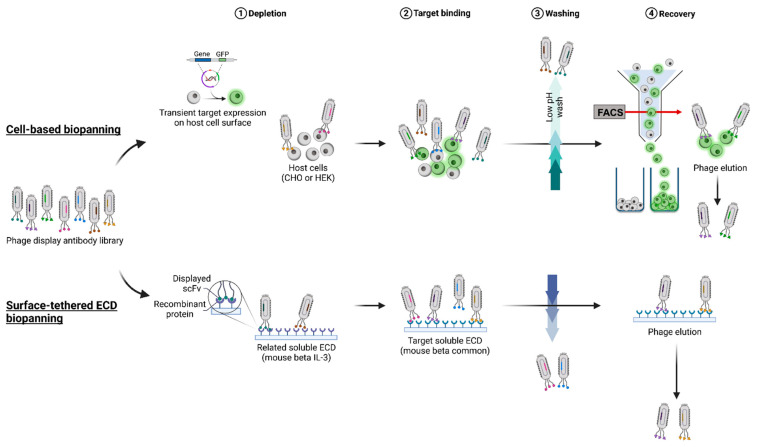
Schematic overview of STRE and CBA biopanning strategies. Initially, a naïve phage display single-chain antibody library was exposed to the related antigen mβ_IL-3_ (STRE biopanning) or cells devoid of the target receptor (CBA biopanning) in a depletion step. Next, remaining phages were allowed to bind to the target antigen (mβc), and non-specifically bound particles or those with low affinity were eliminated by a low pH washing step. Finally, target-specific bound phages were recovered by elution.

In this study, a type I membrane protein, the mouse beta common (mβc) receptor, was used as a model membrane protein to compare two commonly used antigen presentation formats, cell surface and surface-tethered recombinant ECDs, according to their suitability to isolate binders from a naïve single-chain Fv (scFv) fragment antibody library [11]. The mβc receptor belongs to the type I cytokine receptor family and is co-expressed with one or more α-chains (IL-3Rα, IL5-Rα, or GM-CSFRα) on the surface of myeloid cells [12,13]. IL-3, IL-5, and GM-CSF bind to βc at the same interaction site, in an elbow or pocket region that forms at the interface of domain 1 and domain 4 of the homodimeric receptor complex. Interaction with βc further requires a cytokine-specific α-subunit to be present [14,15]. Mutational, structural, and modeling studies have identified several sites in the βc receptor that may be exploited to block cytokine-mediated signaling. These include surface areas important for cytokine binding and other protein–protein interaction sites essential for the formation of a functional dodecameric receptor complex [15]. A panel of 30 novel mAbs was isolated from both STRE and CBA presentation strategies (Figure 1), reformatted into full-length immunoglobulins (mouse IgG2a), and purified for downstream analysis. Purified mAbs were extensively characterized regarding their physicochemical properties, including oligomerization and aggregation behavior, thermal stability, binding, and glycosylation status, followed by an assessment of their functional capacity to block mIL-3-mediated activation of mβc signaling. Only one mAb isolated from biopanning employing the surface-tethered recombinant ECD significantly reduced IL-3 induced STAT5 phosphorylation in a dose-dependent manner. These results provide a better understanding of the effects of different antigen presentation formats and how these strategies may influence the selection of mAbs when certain functional properties are desired.

## 2. Results

### 2.1. Isolation of mAbs Specific to mβc

Two distinct biopanning strategies were pursued to identify clones that bound specifically to the target antigen. One biopanning strategy employed the recombinant mβc ECD (STRE), while transiently transfected cells expressing the full-length mβc at the cell surface were used for the CBA biopanning approach. For STRE, a naïve phage library was screened using the entire recombinant extracellular region of the mouse βc homodimer containing a C-terminal hexa-His-tag. To facilitate the isolation of mAbs unique to mβc and additionally to reduce the incidence of clones that recognize the C-terminal His tag, the recombinant ECD of the related mouse β_IL-3_ was used for negative selection. For CBA, biopanning against mβc displayed on the cell surface (biopanning rounds cR1-cR3) required the transient expression of the mβc receptor in host cells that do not endogenously express mβc. Two mammalian cell lines (CHO and HEK cells) were transfected with an expression construct encoding mβc with GFP fused to the C-terminus (mβc-GFP) to allow detection using flow cytometry and immunofluorescence microscopy. CHO or HEK cell mβc-GFP transfectants were alternated between biopanning rounds to reduce the incidence of phage clones that bound non-specifically to the cell surface. The highest expression of mβc-GFP was observed at 48 h post-transfection, and hence this was determined to be the optimum time point to commence biopanning (Figure S1). Cells transfected with mβc-GFP were also sorted by flow cytometry (Figure S2) to isolate cells potentially binding phage specific to the target antigen. The output titers for both strategies were found to be highest in the final, third biopanning round, indicating enrichment of positive phage (Table S1).

**Figure 2:**
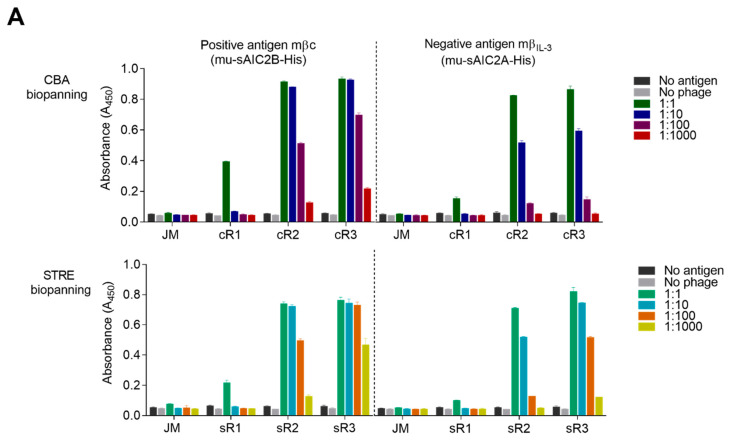

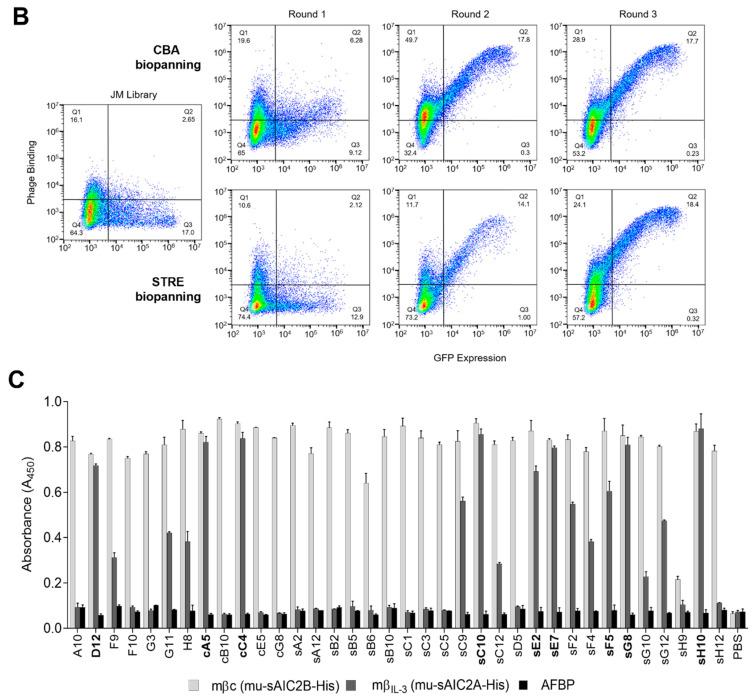
Binding analysis of STRE and CBA biopanning derived phage pools and individual phage clones. (**A**) Polyclonal phage ELISA against the recombinant antigens mβc (mu-sAIC2B-His, positive) and mβ_IL-3_ (mu-sAIC2A-His, negative). Phage pools from all three CBA (cR1-cR3) and STRE (sR1-sR3) rounds were compared to the naïve Jones-Mahler (JM) library. Binding was assessed in duplicates by immobilizing the recombinant proteins on ELISA plates followed by incubation with successive 10-fold dilutions of phage particles. (**B**) Polyclonal flow cytometry analysis of the original phage library and subsequent biopanning pools against transiently expressed mβc-GFP on the surface of CHO-XL99 cells. Protein expression was monitored using the intracellular GFP (*x*-axis), while phage binding was determined using a primary mouse anti-M13 antibody in combination with a secondary anti-mouse IgG antibody conjugated to DyLight650 (*y*-axis). (**C**) Monoclonal phage ELISA of selected purified phage clones that exceeded an A_450_ reading of 0.6 in a preliminary screen (Figure S3). Adipose fatty acid binding protein (AFBP) was used as a non-specific His-tagged negative control. Prefixes ‘c’ and ‘s’ denote clones derived from CBA and STRE biopanning methods, respectively. Clones with no prefix were identified in both biopanning campaigns.

To assess whether these positive phages were specific for the target antigen, phage pools from all biopanning rounds were screened in polyclonal phage enzyme-linked immunosorbent assays (ELISA) (Figure 2a) against the mβc ECD and further analyzed by flow cytometry against cell surface expressed mβc (Figure 2b). While the level of enrichment of target-specific binders is comparable between strategies, flow cytometry analysis revealed further enrichment only between R2 and R3 of STRE but not CBA biopanning (Figure 2b). This is evident by a larger number of recorded events in Q2 between sR2 and sR3, while there is essentially no increase in events in Q2 between cR2 and cR3. However, a large proportion (49.7%) of GFP negative cells (Q1) in cR2 were positive for phage binding, indicating many non-specific cell-surface binders were present in the cR2 pool. Although considerable enrichment of desired clones was observed in cR2, three biopanning rounds were necessary to remove many of the non-specific phages. Additionally, there was a higher incidence of cross-reactive clones recognizing mβ_IL-3_ in the STRE biopanning strategy despite including specific depletion steps. Cross-reactivity was further confirmed by analyzing individual candidates (Figure 2c). In total, 182 clones from both biopanning strategies were isolated and assessed by monoclonal ELISA (Figure 2c and Figure S3). Based on our previous experience with clones isolated from this library, an internal cut-off of an absorbance reading of A_450_ > 0.6 was applied to identify 79 and 68 positive binders from sR3 and cR3, respectively. Amongst these, 16 clones from sR3 were cross-reactive towards mβ_IL-3_, compared to 7 clones isolated from cR3.

Based on CDR-H3 sequences, 28 unique clones were derived from STRE sR3, and 12 unique sequences were identified within the CBA cR3 pool (Table S2). There were 7 clones isolated from both strategies, resulting in a total of 33 positive clones.

### 2.2. Expression and Purification of Full-Length Immunoglobulins

All 33 scFvs were reformatted into full-length mAbs (mIgG2a), transiently transfected into CHO cells, and purified via protein A affinity chromatography from cell culture supernatants. Three candidates (D12, F9, and sH10) failed to express and were excluded from subsequent analysis (data not shown). Purified antibodies migrated at the expected molecular weight (*M*_W_) by SDS-PAGE, with one predominant band at ~150 kDa under non-reducing conditions and two separate bands at ~25 kDa and ~50 kDa under reducing conditions, suggesting the correct association of heavy and light chains (Figure S4). Protein yields of individual clones post-purification varied, ranging between 450 mg/L (G11) and 19 mg/L (cE5). Each purification batch was further screened for the presence of endotoxin and was found to be below the detection limit of the assay (<0.6 EU/mg).

### 2.3. Aggregation Analysis by Analytical SEC

**Figure 3:**
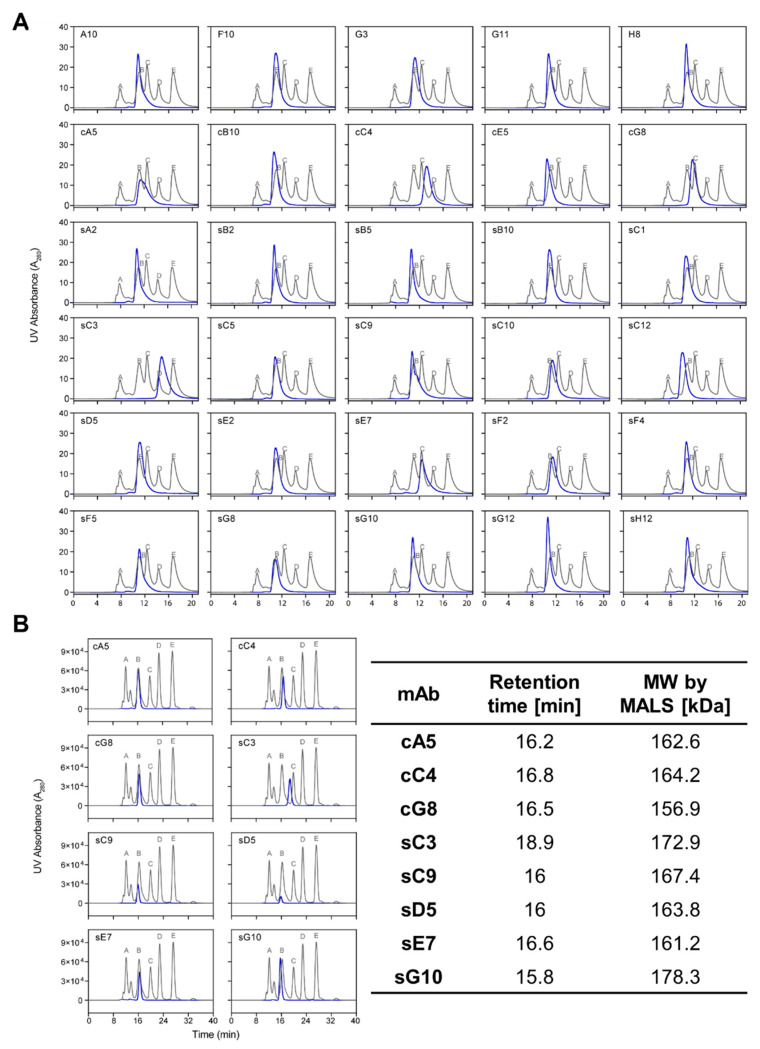
Analytical size exclusion chromatography (SEC) of purified anti-mβc mAbs. Chromatograms show the elution profile of each mAb (blue) overlayed on a standard run of proteins with known molecular weights (*M*_W_) (grey). (**A**) All 30 candidates eluted in a single peak indicative of a homogenous species of molecules in solution. However, six antibodies (cA5, cC4, cG8, sC3, sC9, and sE7) exhibited longer retention times than expected for a full-length IgG molecule when analyzed on a silica-based column. (**B**) These clones were further assessed on a Superdex 200 column coupled to a MALS detector for accurate *M*_W_ determination. Retention times for all antibodies except sC3 were comparable to the IgG protein in the standard (peak B), and *M*_W_s corresponded with theoretically calculated values. Standard proteins: A—thyroglobulin (670 kDa), B—IgG (158 kDa), C—ovalbumin (44 kDa), D—myoglobin (17 kDa), E—vitamin E12 (1.35 kDa).

The aggregation profile of all mAbs was assessed by analytical size exclusion high-performance liquid chromatography (SE-HPLC). A single dominant peak was observed for all mAbs indicating no aggregation or fragmentation. The peak was at the expected retention time for all except six candidates (cA5, cC4, cG8, sC3, sC9, and sE7), which eluted at a higher retention time (Figure 3a). These six antibodies were further analyzed on a size exclusion chromatography (SEC) column with a different stationary phase (Superdex 200), coupled to a multiangle light scattering (MALS) detector (Figure 3b). This allowed accurate determination of absolute molar masses and the average size of each molecule based on scattered light intensity. All analyzed samples except for sC3 were observed at the appropriate retention times. MALS confirmed *M*_W_s for all analyzed full-length IgGs to be within a range of 157–178 kDa.

Thus, longer retention times for some candidates may be due to non-specific interaction with the stationary phase of both SEC columns used in these experiments.

### 2.4. Thermal Stability of Purified mAbs

The thermal stability of anti-mβc mAbs was determined using differential scanning fluorometry (DSF), a fluorescence-based assay that gradually increases the temperature to initiate protein unfolding, which corresponds to fluorescence intensity. The thermal transition midpoint (Tm) is the temperature at which 50% of a protein is folded while the other half is in an unfolded state. The average Tm of all tested mAbs was 63 °C, spanning a range of 20 °C (Table S3). The mAb with the highest Tm was sH12 at 70 °C, and the lowest was sF5 at 50 °C. All mAbs were within the appropriate Tm range described for recombinant antibodies [16], indicating the absence of sequence-induced thermal stability issues.

### 2.5. Glycan Analysis of mAbs by Mass Spectrometry (MS)

Glycosylation profiles of transiently expressed proteins can vary between different batches, even if the same cell line is used [17,18]. As the 30 mAbs were transiently expressed in batches over the course of several weeks, a mass spectrometry analysis was performed to determine the degree and homogeneity of glycosylation on all purified candidate antibodies. We detected the presence of both *N*- and *O*-linked glycosylation sites on the mIgG2a backbone, in agreement with previous reports [19,20]. As expected, *N*-linked glycans were identified at Asn322 within the tryptic peptide E_319_DYN_322_STLR_326,_ which is derived from proteolytic digestion of the Fc domain. This site was predominantly occupied in all analyzed mAbs by 2 monosaccharide compositions, HexNAc_3_Hex_3_Fuc_1_ and HexNAc_4_Hex_3_Fuc_1_, with an average relative abundance of 24.8% and 74%, respectively (Figure S5a). Three additional glycoforms (HexNAc_2_Hex_1_, HexNAc_4_Hex_3,_ and HexNAc_4_Hex_4_Fuc_1_) were also identified, albeit with lower relative occupancy. Representative higher-energy collision dissociation (HCD) mass spectra depicting the different glycoforms at Asn322 are shown in Figure S6.

In addition, several *O*-linked glycoforms were identified in the hinge region of all mAbs, specifically at Thr247 in the peptide G_245_PT_247_IKPCPPCK_255_, albeit at low occupancy (an average of 1.18% occupied). *O*-glycosylation at Thr247 has been described previously for both mouse IgG2a and IgG2b subtypes [19,20]. The most predominant *O*-glycan in all samples at Thr247 was HexNAcHexNeuAc, with an average relative occupancy of 1.67% (Figure S5b). Interestingly, mAb sC3 displayed the highest abundance of this glycoform, with an average occupancy of 24%. This level of occupancy was considerably higher than what was observed for any other clone, with G3 displaying the second highest occupancy at 1.37%. Representative HCD spectra confirming the presence of *O*-linked glycoforms are shown in Figure S7.

Additional *N*-linked and *O*-linked glycosylation sites were identified in CDR1 of the variable heavy chain of clone sC12 within the peptide T_43_SGYN_47_FT_49_SYAMHWVR_57_. In total, seven glycoforms were assigned to this peptide, including the *O*-linked monosaccharide HexNAc at Thr49 and several *N*-linked glycoforms attached to Asn47. This sequon was partially occupied, and the most relatively abundant glycoform identified at this site was HexNAc_4_Hex_3_Fuc_1_ (Figure S8). Moreover, low levels of sialic acid (NeuAc) were observed. HCD mass spectra confirming the presence of *N*- and *O*-linked glycoforms are depicted in Figure S9a–k.

Calculations of relative abundances of all detected glycans can be found in Supplementary File S2.

### 2.6. Binding Analysis of mAbs to the Target Antigen

The binding of anti-mβc mAbs to the target antigen was validated using the soluble mβc ECD in ELISA and surface plasmon resonance (SPR) assays, as well as the native mβc receptor expressed on the surface of NFS-60 cells. As loss of affinity during the reformatting of scFv fragments to IgGs is not uncommon [21,22], all 30 clones were initially subjected to indirect ELISA analysis (Figure S10). Cross-reactivity with mβ_IL-3_ was retained by clones cA5, cC4, sC10, sE7, and sG8 and all clones reacted with mβc upon reformatting.

Next, we determined the real-time binding and dissociation kinetics of the mAbs with mβc in multicycle kinetic SPR experiments, capturing the polyhistidine-tagged mβc ECD via immobilized anti-His antibodies on the surface of CM5 sensor chips and using purified mAbs as analytes. As K_D_ calculations are not accurate due to the multivalent nature of the mAbs and the homodimeric antigen, we limited the analysis to comparing binders according to their off-rates (k_d_) (Figure S11 and Table S4). As the decision of which partner to use as ligand or analyte may affect association and consequently lead to abolished or non-ideal binding, assays were additionally performed in the reverse orientation, capturing mAbs on a Protein A chip and probing the functionalized surface with mβc as analyte. However, no improvements were observed (data not shown).

To confirm specific binding to mβc in the native environment of the cell surface, positive mAbs were assessed by flow cytometry against NFS-60 cells constitutively expressing the mβc receptor subunit. Mouse myeloid leukemia M1 cells that do not express mβc and an unrelated mouse IgG2a mAb were used as negative and mAb isotype controls, respectively. Initially, mIL-3Rα, mβc, and mβ_IL-3_ expression was verified by flow cytometry using commercial antibodies (Figure S12). As expected, cell staining was positive for mIL-3Rα and mβc, but only negligible levels of mβ_IL-3_ were detected.

**Figure 4:**
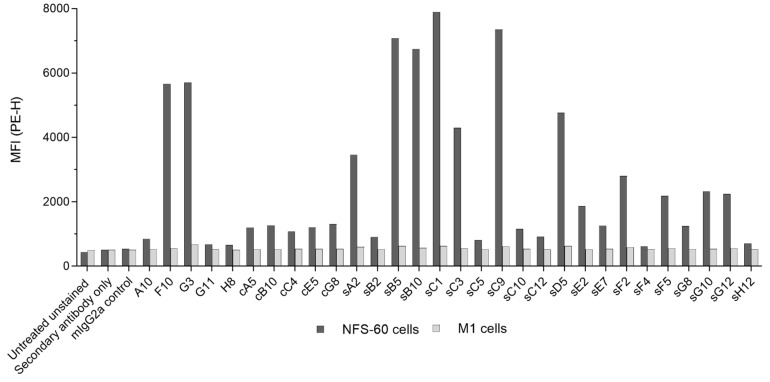
Flow cytometric assessment of purified mAbs binding to NFS-60 cells (dark grey) natively expressing the mβc receptor. Mouse M1 cells (light grey), which do not express mβc, as well as the PE-conjugated secondary antibody and an unrelated mIgG2a isotype were used as negative controls. Cells were incubated with 200 μg/mL of each mAb, and binding is shown as a measure of the mean fluorescence intensity (MFI). Despite being isolated from the CBA biopanning method, corresponding mAbs (prefix ‘c’) generally produced a less pronounced shift indicative of a lower binding affinity for the target molecule in the native cell surface environment. Prefix ‘s’—STRE biopanning method; no prefix—binders isolated from both methods.

Next, using mean fluorescence intensity (MFI) as a measure of binding, a high degree of variability in cell staining was observed across all 30 mAbs (Figure 4). While clones sB5, sB10, sC1, and sC9 produced the most pronounced shift, values for candidates G11, H8, sF4, and sH12 were found to be only marginally above the isotype control. Taking ELISA results into account (Figure S10), it becomes apparent that several clones may recognize the soluble mβc ECD but fail to bind to the membrane-bound full-length mβc. Interestingly, many mAbs derived from CBA biopanning only weakly associated with NFS-60 cells, with the notable exception of clones common to both STRE and CBA strategies, F10 and G3. Lack of binding to M1 cells confirmed the specificity of the mAbs towards the target.

### 2.7. Functional Cell Signaling Inhibition Assays

**Figure 5:**
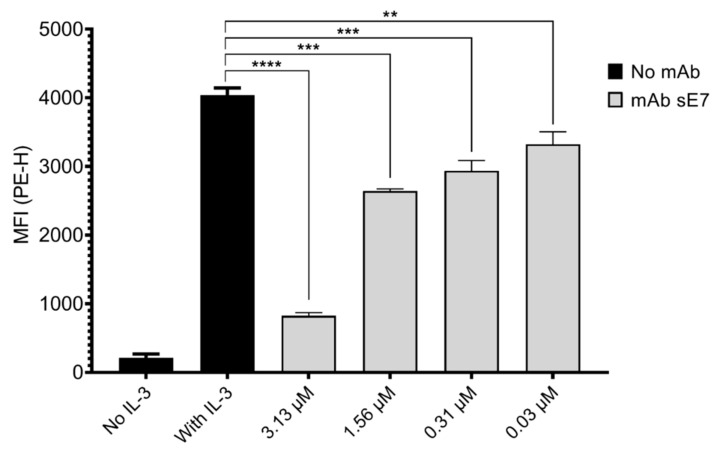
Flow cytometric analysis of pSTAT5 levels in NFS-60 cells following incubation with varying concentrations of mAb sE7 in response to mouse IL-3. Bars represent the mean fluorescence intensity (MFI) of PE after background (unstained cells) subtraction. Unstimulated cells or cells stimulated with 2 ng/mL of mIL-3 are shown as baseline and fully activated controls, respectively. The assay was performed in duplicate (*n* = 2). Error bars represent the SD of two independent experiments. A One-Way ANOVA with a Tukey’s multiple comparisons test was performed to determine significance (**** *p* < 0.0001; *** *p* < 0.001; ** *p* < 0.01).

NFS-60 cells phosphorylate the transcription factor STAT-5 in response to IL-3, a process that is dependent on the presence of mβc and its co-receptor IL-3Rα [15]. We assessed whether any of the candidate mAbs were able to specifically block cytokine-mediated signaling through mβc by measuring phosphorylation of STAT5 (pSTAT5) by flow cytometry. Initially, NFS-60 cells were incubated with increasing concentrations of each antibody and then stimulated with IL-3. Candidates that led to a reduction of pSTAT5 levels of more than 20% in comparison to the control were analyzed further (sC5, sC9, sC10, sC12, sE7, sF4, sG10, sG12, andtable sH12—Figure S13). Surprisingly, only clones derived from the STRE biopanning campaign had an antagonistic effect on mIL-3-mediated signaling. Amongst these nine candidates, only sE7 exhibited a significant decrease in pSTAT5 levels at all tested mAb concentrations in a dose-dependent manner (Figure 5). Reduction of pSTAT5 levels was found in the range of 18–80% for the lowest (0.03 μM) and highest (3.13 μM) mAb concentrations assessed in this study.

### 2.8. Binding Analysis of Mono- and Bivalent sE7

To evaluate whether sE7 would be amenable to further optimization with regards to binding kinetics and overall binding affinity, we directly compared the monovalent scFv and the bivalent mAb in bio-layer interferometry (BLI) real-time binding assays (Figure S14). Ni-NTA biosensors were used to capture the recombinant mβc ECD, and scFvs or mAbs were supplied as analytes in four different concentrations. While association rates (k_a_) were comparable, the dissociation rate k_d_ of the monovalent scFv was more than two orders of magnitude faster than those measured for the bivalent mAb. As a result, the overall affinity (K_D_) of the scFv was relatively weak at 0.324 μM.

## 3. Discussion

The discovery of novel mAbs with high affinity and specificity for a particular target is a fundamental requirement when developing new biologics and research tools alike. However, beyond the isolation of scFv candidates that merely bind their respective antigen, the recognition of well-defined epitopes is often needed for these candidates to exert agonistic or antagonistic effects when employed as therapeutic modalities. Phage display biopanning is an important, widely used tool to identify and isolate specific positive clones from large antibody fragment libraries and develop initial candidates through various further engineering strategies, such as in affinity maturation [23]. The fact that over a dozen mAbs discovered via phage display have been approved for clinical use, with countless more currently being pursued in pre-clinical and clinical phase pipelines, has corroborated its usefulness at the frontline of therapeutic antibody discovery. However, phage display is a multifaceted and often complex process, including several key steps that require careful optimization to achieve isolation of potent mAbs [2]. In addition to the design of the antibody library, the selection and presentation of the antigen remains one of the most critical features that will inevitably impact the quality and suitability of generated clones for a given purpose. Ultimately, the preservation of the native structure, as well as the accessibility and relative abundance of epitopes crucial for the function of a target protein, will determine whether antibodies with the ability to interfere with these functions can be isolated. Membrane proteins represent a considerable number of pharmacological targets [24] and have long posed a challenge as they are difficult to purify and maintain in their native conformations for conducting a biopanning campaign. To overcome these challenges, several approaches can be pursued, including the use of recombinant ECDs [25] and cell lines that heterologously express the full-length protein on the cell surface [11,26]. In comparison to biopanning campaigns employing a soluble recombinant receptor ECD, whole cell biopanning protocols are often more complex, and screening to eliminate non-specific cell-surface binders can be laborious and time-consuming. However, truncation of a membrane protein into a soluble form may lead to the exposure of non-native and otherwise non-accessible epitopes, which may result in a substantial increase in diversity of the final phage pool. Consequently, sequences that recognize a certain epitope with functional importance can be considerably less frequent in the final phage pool and thus a much larger number of clones may need to be screened in functional assays to identify a potent inhibitor. While both strategies have been successful in generating binders with therapeutic potential, there is a lack of comparative studies with regards to antigen presentation within the context of the native cell surface proteome and environment to drive antibody selection towards a specific epitope required for ligand-induced effector function.

In this study, we used mβc as a model antigen to explore whether surface-tethered recombinant ECDs are more suitable than cell-based biopanning strategies to generate binders with functional potential. We assessed a panel of 30 antibodies isolated from both methods with regard to their ability to block cytokine-mediated activation of the receptor. The STRE biopanning strategy yielded a higher proportion of positive binders (87%) compared to the CBA strategy (75%) and produced approximately twice as many unique sequences. Moreover, half of all unique CDR-H3 sequences found in pool cR3 were identical to those present in the sR3 phage pool. Out of the 33 unique scFv sequences we reformatted into mIgG2a backbones, 3 clones failed to express, 2 of which were derived from both biopanning methods. The reason for this lack of expression remains elusive as we did not detect any significant sequence liabilities in terms of CDR length and hydrophobicity, overall hydrophobicity, calculated isoelectric points (pI), or predicted glycosylation sites (data not shown). A higher aggregation propensity, incorrect chain assembly, or impaired folding of the variable domains may constitute plausible reasons for the hampered secretion of these difficult-to-express molecules [27,28]. SDS-PAGE, analytical SEC and thermal stability analysis of the remaining 30 candidates revealed the presence of stable, correctly assembled, and highly pure mAbs that eluted in a single peak indicative of homogeneous preparations devoid of non-native aggregates (Figure 3). This was further confirmed by absolute molecular weight measurements of selected candidates by MALS. As glycans represent important post-translational modifications (PTMs) with the potential to impact stability, potency, and binding affinity of mAbs, glycosylation profiles were determined by mass spectrometry to discern any significant differences between candidates. The *N*- and *O*-linked glycosylation sites determined in all analyzed mAbs were located on the expected sequences in the Fc and hinge regions and have been extensively characterized [29,30,31,32]. In addition, glycans have also been identified in the variable regions of approximately 10% of mAbs [33]. These glycans may either increase or decrease the affinity towards a particular antigen, thereby modulating the functional activity of mAbs. As glycans can have a profound impact on the production and secretion of proteins, variable region glycans are often considered a sequence liability in the bio-manufacturing industry [34]. The presence of sequons in the variable region was predicted for mAbs A10 and sC12 based on the amino acid sequence, but glycosylation at these sites could only be confirmed for sC12 (Figures S8 and S9). Possible reasons to explain the lack of glycosylation at the predicted site in A10 include negative impacts on biosynthesis, occupancy below the limit of detection, the presence of other PTMs in the peptide, or suboptimal sample preparation and MS analysis conditions or parameter. In contrast, several *N*-linked glycans were observed at the variable region in sC12. For example, the monosaccharide HexNAc could be assigned to a peptide originating from V_H_, which comprises part of CDR-H1 (Figure S9). No glycans could have been present when isolating the scFv fragment from the bacterially expressed library, and in this case, glycosylation at these positions in the mammalian expressed mAbs did not appear to have a major impact on antigen binding.

Receptor recognition and binding was confirmed for all 30 IgG clones by ELISA, and apparent dissociation constants (k_d_) were determined to rank clones according to their relative dissociation affinities for the target molecule using SPR. Several clones failed to bind to mβc or displayed poor binding behavior (e.g., sC10 and cB10) in the microfluidics environment of SPR sensor chips regardless of whether mAbs were used as ligands (Fc capture on the surface via Protein A) or as analytes (anti-His capture of the tagged mβc ECD). Lack of binding may be explained by steric occlusions of specific epitopes or protein aggregation within the flow cells [35]. The non-ideal binding behavior of some clones may further be due to the bivalent nature of full-length IgG molecules. Candidates that produced sensorgrams demonstrated dissociation rate constants (k_d_) suggestive of nanomolar binders (Figure S11). However, it is important to note that these results do not reflect absolute affinities of the evaluated mAbs as the two epitope binding sites of the individual antibodies result in increased avidity for the target. Flow cytometry analysis revealed that all clones were able to recognize mβc constitutively expressed on the surface of NFS-60 cells, albeit to varying degrees (Figure 4). However, most mAbs (including the entire cell-based mAb panel and more than half of the clones derived from both biopanning strategies) produced only a small shift in mean fluorescence intensity (MFI), while the majority of mAbs exclusively discovered via the STRE method bound to cell surface mβc more effectively than the CBA biopanning derived mAbs. This is somewhat surprising as biopanning against mβc on the surface of cells was expected to favor selection of scFvs that bind to epitopes more readily accessible within the cellular environment. No correlation between relative dissociation rates with the degree of cell binding was observed (data not shown), suggesting that the targeted epitopes may be the reason for these results. These epitopes may have been occluded by the formation of transient low-affinity complexes of mβc with IL-3Rα (Figure S12), IL-5Rα, or GM-CSFRα on the surface of NFS-60 cells. However, the formation of functional high-affinity receptor complexes requires the presence of the respective cytokines, which were not supplemented during cell surface binding experiments. However, these epitopes would have also been accessible on the recombinant soluble ECD during STRE biopanning together with other, non-native epitopes that resulted from the removal of the transmembrane domain. Thus, the lower sequence diversity obtained from CBA biopanning in combination with a focus on epitopes that are rather inaccessible on the surface of NFS-60 cells may have contributed to the weaker binding as observed for CBA-derived mAbs.

The ability of anti-mβc mAbs to inhibit IL-3 mediated STAT-5 phosphorylation candidates was tested by a flow cytometry assay measuring pSTAT5 levels in NFS-60 cells first starved of and later stimulated with the cytokine. Only one clone (sE7), which was derived from the STRE biopanning campaign, showed significant dose-dependent inhibition of receptor activation at all tested concentrations (Figure 5). A relatively high concentration of 3.13 μM (500 μg/mL) was required to decrease pSTAT5 levels by 80%, and we thus asked whether this clone may be amenable for affinity maturation. Consequently, we determined kinetic constants (k_a_ and k_d_) and the K_D_ of the monovalent scFv, which displayed a dissociation constant two orders of magnitude higher than the corresponding mAb (Figure S14). Thus, dissociation of sE7 from the ligand could be further improved [36,37] as slow dissociation rates constitute an important characteristic for therapeutic antibodies to increase efficacy at lower doses [35]. The direct comparison of the monovalent scFv and the bivalent mAb further illustrates the impact of avidity on overall K_D_.

In view of the numerous strategies employed for biopanning, it is surprising that relatively few comparative studies exist examining the impact of antigen presentation with respect to membrane proteins. In a recent example, four different biopanning strategies, including the immobilization of recombinant protein to polystyrene beads and different cell-based methods, evaluated the selection output when incorporating various depletion and blocking steps [38]. Lakzaei and colleagues compared the effects of an antigen in solution to the same antigen immobilized on a carrier surface and found that the soluble approach was more efficient for isolating functional antibody fragments against the target [39]. However, comparative studies reported in the literature mainly focus on the optimization of strategies, including pH-elution parameters [39,40], the impact of depletion and negative selection [41], and the use of different antigen capture reagents [42,43,44]. Here, we show that biopanning strategies employing the surface-tethered recombinant ECD of an integral type I membrane protein were more effective in isolating a functional binder compared to biopanning with the full-length protein in the cellular membrane environment. This result can be mainly attributed to the higher clonal diversity which appears to be beneficial over targeting a limited set of epitopes. Work is currently underway to fully capture the genetic diversity of the isolated antibody repertoire, which will further inform optimal antibody discovery methods for therapeutically relevant cell surface targets.

## 4. Materials and Methods

Details for bacterial strains used in this work are provided in Table S5.

### 4.1. Antibody Reagents

PE-conjugated rat anti-mouse CD131 (mβc) antibody (BD Pharmingen, cat# 559920, RRID: AB_397374); Mouse anti-M13 antibody (SinoBiological, Beijing, China; cat# 11973-MM05T, RRID: AB_2857926); HRP conjugated goat anti-mouse IgG antibody (BioRad, South Granville, NSW, Australia; cat# 1706516, RRID: AB_11125547); DyLight 650 conjugated goat anti-mouse IgG antibody (Abcam, Melbourne, VIC, Australia; cat# 96874, RRID:AB_10679531); Isotype mouse IgG2a control antibody (Miltenyi Biotec, Macquarie Park, NSW, Australia; cat# 130-106-546, RRID:AB_2661589); PE-conjugated mouse anti-STAT5 (pY694) antibody (BD Biosciences, Macquarie Park, NSW, Australia; cat# 612567, RRID:AB_399858); PE-conjugated rat anti-mouse CD123 (IL-3Rα) antibody (eBioscience, San Diego, CA, USA; cat# 12-1231-82, RRID:AB_465839); AF488 conjugated rat anti-mouse β_IL-3_ antibody (R&D Systems, Minneapolis, MN, USA; cat# FAB5492G, RRID: RRID:AB_2905558); Rat anti-mouse CD131 (mβc) antibody (BD Biosciences, cat# 740050, RRID:AB_2739817).

### 4.2. Cell Lines

Chinese hamster ovarian cells (CHO) XL99 (Acyte Biotech, Brisbane, QLD, Australia) and Freestyle293 human embryonic kidney cells (HEK) (Thermo Fisher Scientific, Seventeen Mile Rocks, QLD, Australia) suspension cultures were maintained, shaking at 37 °C with 7.5% CO_2_, in CD-CHO (Gibco, Thermo Fisher Scientific) and Freestyle293 (Gibco, Thermo Fisher Scientific) media respectively, containing 8 mM GlutaMAX (Gibco, Thermo Fisher Scientific) and supplemented with 0.4% anti-clumping agent (ACA) (Gibco, Thermo Fisher Scientific). Cells were passaged every 3–4 days, and 24 h prior to transfection, cells were seeded without ACA. ExpiCHO (Thermo Fisher Scientific) cells were maintained, shaking at 37 °C with 7.5% CO_2_ in ExpiCHO Expression Medium (Gibco), with routine passaging every 3–4 days.

M1 cells were a kind gift from Associate Professor Barbara Rolfe (AIBN, UQ, Brisbane, QLD, Australia). NFS-60 and M1 cells were grown in RPMI 1640 media (Sigma-Aldrich, Castle Hill, NSW, Australia) supplemented with 10% fetal bovine serum (FBS, Sigma-Aldrich), 500 U/mL Penicillin–500 µg/mL Streptomycin (Pen-Strep, Gibco, Thermo Fisher Scientific), and 20 mM GlutaMAX (Gibco, Thermo Fisher Scientific),). Media for NFS-60 cells was supplemented with 1 ng/mL of mouse IL-3 (R&D Systems), and cells were passaged every 2 to 3 days using a 1:50 dilution. M1 cells were passaged at a 1:10 dilution every 3 to 4 days. Cells were maintained at 37 °C, 5% CO_2_ in a humidified incubator.

### 4.3. Phage Display Biopanning Using the Recombinant mβc ECD

Three rounds of biopanning were performed as described previously [45]. Briefly, the recombinant ECDs of mβc amino acids 30–445 (Genbank Accession number NP_031806.3), including a C-terminal hexahistidine tag and mβ_IL-3_ amino acids 30–444 (Genbank Accession number NP_031807.1) including a C-terminal hexahistidine tag were provided by CSL Limited and used as positive and negative antigen, respectively. Purified antigens were coated onto Nunc-Immuno tubes overnight at a concentration of 10 µg/mL in STRE biopanning round 1 (sR1). For subsequent rounds (sR2-sR3), the concentration of positive antigen mβc was reduced to 5 µg/mL. Coated tubes were washed 3 times with phosphate-buffered saline (PBS) and blocked, rotating for 1 h with 2% milk-PBS (MPBS). Simultaneously, 1 mL (10^13^ cfu/mL) of an in-house naïve human scFv library (Jones-Mahler library) provided by the National Biologics Facility (NBF) [11] was blocked using a final concentration of 2% MPBS for 1 h rotating at room temperature (RT). Blocked phage particles were added to negative antigen-coated tubes for depletion. Two depletion steps were performed per round for 1 h each with rotation at RT. Depleted phage pools were added to the positive antigen-coated tube and incubated for 1 h with rotation before being discarded. The tubes were rinsed with PBS, including 0.1% Tween 20 (PBST) followed by PBS. The number of washes was increased for stringency from 3 times for sR1 to 10 times for sR2 and 20 times for sR3. Bound phages were eluted with rotation for 8 min using 1 mL 200 mM glycine, pH 2.5, followed by neutralization with 1 mL 1 M Tris-HCl, pH 7.4. Half of the eluate was used to infect 10 mL of log-phase *Escherichia coli* (*E. coli*) XL1-Blue cells for 30 min at 37 °C, while the other half was made into a glycerol stock for storage at −80 °C. Following infection, cells were collected by centrifugation and grown overnight at 30 °C on 2 150 mm 2X Yeast-Tryptone (2YT)-agar plates supplemented with 100 ug/mL Ampicillin and 2% glucose (2YT-Amp-Glu). The following day, bacterial colonies were retrieved by washing plates with 5 mL 2YT-Amp-Glu, creating a slurry which was then used to inoculate 50 mL bacterial 2YT-Amp-Glu media to an initial OD_600_ of 0.05. *E. coli* were grown at 37 °C, 200 rpm to an OD_600_ of 0.4–0.6, followed by the addition of 1 × 10^11^ M13K07 helper phage particles (New England BioLabs, Notting Hill, VIC, Australia). Cultures were incubated for 30 min at 37 °C, followed by an additional 30 min at 37 °C and 200 rpm before centrifugation at 2000× *g* for 10 min. Cell pellets were resuspended in 100 mL 2YT-Amp supplemented with 50 μg/mL Kanamycin, and cultures were incubated overnight at 30 °C with agitation. Cells were pelleted, and phage particles were precipitated by the addition of PEG-6000 and NaCl to a final concentration of 5% and 500 mM, respectively. Precipitation was carried out at 4 °C for 1 h, followed by centrifugation at 10,000× *g* for 15 min. The phage pellet was resuspended in 10 mL cold PBS and further purified by a second round of precipitation. After centrifugation at 10,000× *g* for 15 min, phage pellets were resuspended in 3 mL PBS supplemented with 20% glycerol and stored at −80 °C for subsequent biopanning rounds.

### 4.4. Cell-Based Biopanning

The CHO codon-optimized full-length mβc gene (GeneArt, Thermo Fisher Scientific) was cloned into pEGFP-N1 (Clonetech, Takara Bio, San Jose, CA, USA) using AgeI and NheI restriction sites, yielding a C-terminal GFP fusion protein. CHO-XL99 (Acyte Biotech) or Freestyle™ 293-F (Gibco, ThermoFisher Scientific) cells were seeded to a density of 3 × 10^6^ cells/mL and transfected with plasmid expression constructs (1 μg/mL cell suspension) with polyethylenimine (PEI-Max, Polysciences, Taipei, Taiwan) at a ratio of 1:4 (*w*/*v*) prepared in OptiPro serum-free medium (Gibco, Thermo Fisher Scientific). Following addition of the DNA complex to the cells, cultures were incubated for 4–6 h, shaking at 37 °C. Cell suspensions were diluted to a final concentration of 1 × 10^6^ cell/mL and further incubated, shaking at either 32 °C (CHO-XL99) or 37 °C (Freestyle™ 293-F) in a humidified incubator for 48 h.

Expression and localization of recombinant mβc-GFP to the membrane of cells was confirmed via immunofluorescence microscopy. Briefly, transiently transfected cells were harvested by centrifugation and resuspended in PBS supplemented with 0.1% sodium azide (PBA). Cells were seeded in a 96-well plate at 2.5 × 10^5^ cells/well, pelleted by centrifugation, and blocked in 25 μL PBA supplemented with 0.1% rat serum for 30 min at 4 °C. Cells were stained with a PE-conjugated rat anti-mouse CD131 antibody at a final dilution of 1:50 for 1 h on ice, followed by 3 washes with PBA. Nuclear staining was achieved with a 1:5000 dilution of Hoechst 33342 (Invitrogen, Thermo Fisher Scientific) during an incubation of 15 min on ice, followed by 3 washes with PBA. Cells were resuspended in PBA and deposited onto Superfrost Plus slides (Thermo Fisher Scientific) at 500 rpm for 5 min with medium acceleration. Slides were dried at 37 °C for 1 h, and coverslips were mounted using ProLong Diamond Antifade Mountant (Invitrogen, Thermo Fisher Scientific). Fluorescence microscopy was performed on an Olympus BX61 microscope.

CBA biopanning was performed as previously described [11]. In brief, transfected cells were used 48 h post-transfection and alternated between 3 rounds, with the first and last selection rounds (cR1 and cR3) performed using CHO-XL99 and the second using Freestyle™ 293-F (cR2). For each round, the library was initially depleted against the corresponding non-transfected cell line. Depletion of non-target-specific cell surface binders is further facilitated by the relatively low efficiency of PEI-mediated transient transfections. CHO and HEK cell surface binders distribute amongst all GFP-negative cells, while FACS sorting of GFP-positive cells maximizes recovery of target-specific phage particles. In total, 10^7^ cells were harvested by centrifugation, washed with 10 mL ice-cold PBS, and blocked in 2% MPBS for 30 min, rotating at 4 °C. Simultaneously, 1 mL of the naïve human scFv phage library (10^13^ cfu/mL) was blocked as described above. The blocked phage particles and non-transfected cells were combined and incubated for 1 h, rotating at 4 °C. Transfected and non-transfected cultures were centrifuged, and the transfected cell pellet was resuspended in the supernatant from the non-transfected cells (containing the unbound phage particles). The suspension was incubated for 1 h, rotating at 4 °C, after which phage-bound cells were washed 3 times by centrifugation and resuspension of the pellet in 5 mL PBS-citrate, pH 5, 0.1% Tween 20, followed by 3 washes with PBS. Cells expressing high levels of GFP were collected by fluorescence-activated cell sorting (FACS) using a FACSAria Cell sorter (BD Biosciences). Sorted cells were centrifuged, and cell-bound phages were eluted with 500 µL 75 mM citric acid, pH 2.3 for 6 min, followed by neutralization with 500 µL 1 M Tris-HCl, pH 7.4. The eluate was used to infect 10 mL of log-phase *E. coli* XL1- Blue for 30 min at 37 °C. Following incubation, phage-infected bacteria were collected by centrifugation and grown overnight at 30 °C on a 150 mm 2YT-Agar-Amp-Glu plate. Phage rescue and amplification was performed as described for STRE biopanning rounds.

### 4.5. Polyclonal ELISA of Phage Pools

The binding reactivity of each phage pool to the surface-tethered recombinant ECD was tested in a 96-well ELISA plate (Nunc MaxiSorp, Thermo Fisher Scientific) coated overnight with 10 µg/mL of purified mβc (mu-sAIC2B-His) or mβ_IL-3_ (mu-sAIC2A-His), followed by 3 washes with PBS. Antigen-coated wells and all phage pools, including the original phage scFv library, were blocked with 400 µL 2% MPBS. Next, 200 µL of blocked phage particles were added in duplicate, then serially diluted to 1:1000 across wells and left to incubate for 1 h. Wells were washed 3 times with PBST followed by incubation with a 1:5000 dilution of an anti-M13 mouse antibody for 1 h. Wells were washed 3 times with PBST and incubated with an HRP-conjugated goat anti-mouse IgG secondary antibody at a 1:5000 dilution for 1 h. Wells were washed 3 times with PBST prior to the addition of 100 µL of 1-Step Turbo TMB-ELISA substrate solution (Thermo Fisher Scientific). After 10 min, the reaction was quenched with 100 µL 2 M sulphuric acid, and HRP activity was detected at an absorbance of 450 nm using the PowerWave X52 microplate spectrophotometer (BioTek Instruments Inc., Currumbin, QLD, Australia).

### 4.6. Polyclonal Flow Cytometry Analysis of Phage Pools

The binding reactivity of each phage pool generated from both biopanning methods was screened against CHO-XL99 cells transiently expressing mβc. Transfected cells were harvested by centrifugation and blocked in 2% MPBS for 30 min, rotating at 4 °C. All phage pools, including the original phage scFv library, were blocked, rotating in 2% MPBS for 1 h. A total of 10^6^ blocked cells were used per sample, pelleted by centrifugation and combined with the blocked phage particles for 1 h, rotating at 4 °C. Cells were washed 3 times with ice-cold PBS, and a primary mouse anti-M13 antibody was added at a dilution of 1:300 for 1 h at 4 °C. Following 3 washes with PBS, cells were stained with a secondary goat anti-mouse DyLight 650 polyclonal antibody (1:500) at 4 °C. Cells were washed 3 times with PBS before analysis on a CytoFLEX Flow Cytometer (Beckman Coulter Life Sciences, Mount Waverley, VIC, Australia). The collected data were processed using FlowJo v10.6.2 (FlowJo LLC, BD Biosciences).

### 4.7. Monoclonal ELISA of Individual Phage Clones

To identify binders specific to mβc, random single *E. coli* clones infected with sR3 and cR3 phage pools were grown on 2YT-Agar-Amp-Glu plates and transferred into liquid cultures in 96-well round bottom plates. Clones were cultured overnight at 37 °C and sub-cultured the following day. Phage production was induced by the addition of 8 × 10^8^ pfu/well M13KO7 helper phage as described above. Phages were amplified overnight at 30 °C in 2YT-Amp-Kan, followed by removal of cells by centrifugation and harvesting of the supernatant containing phage particles. Supernatants from each well and individual wells of 96-well ELISA plates that had been coated overnight with 3 µg/mL either of mβc (positive antigen) or mβ_IL-3_ (negative antigen) were blocked with 2% MPBS for 1 h. Blocked phage particles were added to corresponding wells of the positive and negative antigen-coated plates, and the ELISA was performed as described for the polyclonal phage ELISA.

Positive phage binders were rescreened using purified phage particles, titrating defined concentrations (cfu/mL) in duplicate over 4-fold dilutions starting at 10^10^ cfu/mL. The scFv sequence of positive clones was amplified and sequenced with primers given in Table S6.

### 4.8. Reformatting of scFvs into Full-Length IgGs

All candidate phage clones were reformatted into mouse IgG2a (mIgG2a) using the InFusion cloning system (Clontech, Takara Bio) and a set of mAbXpress vectors, as previously described [46]. These vectors contain constant region sequences derived from Genbank accession AB097847.1 for the G2a heavy chain, BAB33404.1 for the kappa chain, and J00587.1 for the lambda chain. The variable regions were amplified from the purified phagemid vectors using primers outlined in Table S7.

### 4.9. Expression and Purification of Full-Length mAbs

Recombinant mAbs were produced using the ExpiCHO™ Expression System (Gibco, ThermoFisher Scientific) according to manufacturer instructions following the ‘high titer’ protocol. Supernatants of expression cultures were harvested by centrifugation when cell viability dropped below 70% (typically 8–10 days post-transfection). Supernatants were further cleared by filtration using 0.22 µm syringe filter units (Millex Millipore, North Ryde, NSW, Australia) and loaded onto a prepacked 5 mL HiTrap MabSelect SuRe column (Cytiva, Macquarie Park, NSW, Australia) preequilibrated with PBS. Following a wash step with PBS, column-bound proteins were eluted using 0.1 M glycine pH 3. Eluates were immediately neutralized to a pH between 7 and 8 with appropriate volumes of 1 M Tris-HCl, pH 9. The samples were concentrated, and the buffer was exchanged to PBS using Amicon Ultra Centrifugal Filters with a molecular weight cut-off (MWCO) of 50 kDa. Samples were screened for endotoxin using the Charles River Endosafe PTS and the appropriate PTS cartridges with a sensitivity of 0.01 EU/mL, according to manufacturer’s instructions.

### 4.10. Expression and Purification of sE7 scFv

The sE7 scFv was cloned into pET28b with a C-terminal StrepII tag using NcoI and NotI restriction sites. The protein was expressed in *E. coli* Shuffle cells (New England BioLabs) upon induction with 1 mM isopropyl ß-D-1-thiogalactopyranoside (IPTG) in Terrific Broth (TB) medium overnight at 25 °C, 200 rpm. Soluble proteins were extracted into PBS and purified on a prepacked 5 mL StrepTrap column (Cytiva) preequilibrated with PBS. The column was washed with PBS to remove non-specifically bound proteins, and scFvs were eluted using PBS supplemented with 2.5 mM desthiobiotin (Sigma-Aldrich). Elution fractions were concentrated, and the buffer was exchanged to PBS using Amicon Ultra Centrifugal Filters with a molecular weight cutoff of 10 kDa.

### 4.11. Sodium Dodecyl Sulfate–Polyacrylamide Gel Electrophoresis (SDS-PAGE)

Purified mAbs were assessed by SDS-PAGE under reducing and non-reducing conditions. A total of 5 µg of each mAb was combined with an appropriate volume of 4x Laemmli sample buffer (BioRad). To reduce protein samples, 2.5% β-mercaptoethanol was added. Samples were heated to 95 °C, loaded onto 4–15% stain-free gradient gels (BioRad), and run at 200 V for 30 min before imaging on a ChemiDoc system (BioRad).

### 4.12. Indirect ELISA Using Purified Proteins

Purified mAbs were screened by ELISA against recombinant proteins mβc and mβ_IL-3,_ essentially as described for the phage ELISAs above. All mAbs were assessed in a concentration range from 10 µg/mL to 1 ng/mL over a series of 8 dilutions against both antigens coated onto the ELISA plate at 5 µg/mL. Non-linear regression was performed in GraphPad Prism v. 9 (GraphPad Software, San Diego, CA, USA) to produce binding curves.

### 4.13. Analytical Size Exclusion Chromatography (SEC)

Particles were removed from samples by centrifugation at 10,000× *g* for 10 min, and volumes corresponding to 20 µg protein were injected onto a TSK-GEL G3000SWXL column (Tosoh Biosciences, Tokyo, Japan) preequilibrated with PBS supplemented with 200 mM NaCl, pH 6.8. Runs were performed on a 1200 Series HPLC System (Agilent Technologies, Mulgrave, VIC, Australia) at a flow rate of 0.8 mL/min. Calibration was achieved using a gel filtration *M*_W_ standard (Sigma-Aldrich). Selected samples that displayed unusual elution profiles were further analyzed using a Superdex 200 Increase 10/300 GL column (Cytiva) coupled to a multiangle light scattering (MALS) detector at a flow rate of 0.75 mL/min.

### 4.14. Differential Scanning Fluorimetry (DSF)

Thermal stability measurements were performed using the CFX96 Real-Time System and C1000 Thermal Cycler (BioRad). A total protein amount of 10 µg was diluted in PBS to a final volume of 20 µL, and 5 µL of a 50x SYPRO Orange dye (ThermoFisher Scientific) was added to each sample. Measurements were performed in a range of 25 to 95 °C at a heating rate of 1 °C/min. The data were analyzed using CFX Manager 3.1 software (BioRad), and the thermal transition midpoint (Tm) was determined from the first derivative of the unfolding curve.

### 4.15. Mass Spectrometry (MS) Sample Preparation

All mAbs were prepared for proteomic analysis by denaturing the proteins and reducing cysteines by incubating 10 µg of each sample in 6 M guanidinium chloride (GdCl) and 50 mM Tris pH 8.0, with 10 mM dithiothreitol (DTT) for 1 h, shaking at 30 °C. Samples were alkylated by the addition of acrylamide to a final concentration of 25 mM and incubation in the dark, shaking for 1 h at 30 °C. Excess acrylamide was quenched with 5 mM DTT before proteins were precipitated overnight at −20 °C using 4 volumes of methanol/acetone (1:1 v/v). Proteins were pelleted by centrifugation at 18,000× *g* for 10 min, the supernatant was discarded, protein pellets were air dried and reconstituted in 50 µL of 50 mM NH_4_HCO_3_ supplemented with 0.4 µg of trypsin (T6567, Sigma-Aldrich) to achieve a 1:25 enzyme to protein ratio. Samples were incubated at 37 °C with shaking overnight, and digested proteins were cleaned using C18 ZipTips (Millipore). Peptides were reconstituted in 100 µL 0.1% formic acid (FA). All samples were analyzed by reverse-phase LC-MS/MS using a Dionex UltiMate 3000 RSLC nano-system (Thermo Fisher Scientific), with samples separated at a flow rate of 30 µL/min. Desalting was performed on a PepMap 100 C18 trap (5 µm, 0.3 × 5 mm, Thermo Fisher Scientific) for 5 min, followed by separation at a flow rate of 300 nL/min on a Vydac Everest C18 column (5 µm, 150 mm × 75 µm, Hichrom). Peptides were separated for 35 min over a gradient of 5–60% buffer B (80% acetonitrile/0.1% FA) in buffer A (1% acetonitrile / 0.1% FA). Eluted samples were analyzed on an Orbitrap Elite mass spectrometer (Thermo Fisher Scientific) using an NSI electrospray interface. Scans across the *m/z* range of 350–1800 for fragment ions were acquired at a resolution of 30,000 in the Orbitrap. The top 10 precursors were selected for fragmentation by higher-energy collisional dissociation (HCD).

### 4.16. Glycoproteomic Analysis

A focused protein database was first created by combining all candidate mAb protein sequences together with the CHO proteome (UniProt UP000001705, downloaded 14 April 2020 with 23,885 reviewed proteins) and including decoys and common contaminants. Searches were performed using PMBios (Protein Metrics v. 3.8.13, Cupertino, CA, USA) with QTOF and HCD fragmentation selected. The cleavage specificity was set as C-terminal for trypsin (Arg/Lys), and the enzyme specificity was set as fully specific for tryptic digest, with a maximum allowance of one missed cleavage. A mass tolerance of 10 ppm was applied to both precursor and fragment ions, with an *m/z* tolerance of 0.02 Da. Propionamide was set as a fixed modification, and variable modifications were set according to the number and types of modifications predicted on a single peptide. These included oxidation, deamidation, phosphorylation of N-terminal glutamine (Gln) and glutamic acid (Glu), and formylation, all set to “Common 1”. The setting “Rare 2”, which allowed each modification to be present twice on a peptide, included the 6 most common *O*-glycans and 57 human plasma *N*-glycans listed in the PMBios Glycan Databases. Modifications with the *N*-linked monosaccharide composition of HexNAc_3_Hex_3_Fuc_1_ and the *O*-linked HexNAcHexNeuAcNeuGc and HexNAcHexNeuGc compositions were included manually. A maximum of one common and two rare modifications were allowed. All mass spectra for peptides with assigned PTMs were manually inspected, and the best spectrum for each PTM was included in the results. Occupancy for each glycoform at a specific site was calculated by dividing the area under curve (AUC) of each glycopeptide by the AUC of the sum of modified and non-modified peptides. Occupancy at each site was calculated by dividing the summed AUC of all occupied forms of the peptide by the summed AUC of all occupied and unoccupied forms of the peptide (modified from [47]).

### 4.17. Surface Plasmon Resonance Binding Analysis

Multicycle kinetics was performed on a Biacore 8K+ instrument (Biacore Life Sciences, Cytiva) to determine the binding affinities for anti-mβc mAbs. A Series S CM5 sensor chip (Cytiva) was used together with an EDC/NHS amine coupling kit (Cytiva) to immobilize anti-His antibodies to ~13,000 RU on flow cells 1 and 2 as per manufacturer instructions. Commercially available HBS-EP+ buffer (10 mM HEPES, 150 mM NaCl, 3 mM EDTA, 0.05% surfactant P20) was used as running buffer and to dilute the ligand and analyte. Recombinant mβc (1 μg/mL) was captured for 30 s at a rate of 10 µL/min on the surface of flow cell 2, while flow cell 1 was left unmodified and served as the reference surface for subtracting buffer contributions to the binding signal. The soluble common beta ECD containing a C-terminal His tag was used to condition the chip in separate cycles at 1 µg/mL for 30 s with a flow rate of 10 µL/min, prior to ligand capture. MAbs were diluted to 1 nM in HBS-EP+ and assessed using a series of 5 analyte concentrations from 1 nM to 62.5 pM. Association of each analyte was performed over 300 s, followed by dissociation for 600 s. The sensor surface was regenerated between runs using 10 mM glycine pH 1.5 for 60 s at a flow rate of 30 µL/min. Binding assays were conducted at 37 °C. Data were corrected by subtraction of blank runs, and sensorgram curves were fitted using a 1:1 binding model in the Biacore Insight Evaluation Software (Biacore Life Sciences, Cytiva). Dissociation (k_d_) kinetic rate constants were derived based on the local fit at each concentration.

### 4.18. Bio-Layer Interferometry (BLI) Binding Analysis

Binding studies for sE7 were performed by BLI using the full-length mAb and the scFv on an Octet 96 instrument (ForteBio, Sartorius, Dandenong South, VIC, Australia). The sE7 mAb and scFv were diluted in PBS additionally supplemented with 200 mM NaCl and used as the analyte at 4 different concentrations (mAb 12.5–100 nM; scFv 125–1000 nM). A biosensor without captured ligand was used as the reference sensor for subtraction. Binding kinetics were determined using an association time of 100 s and a dissociation time of 400 s. Sensorgrams were processed and fitted using Data Analysis 9.0 software (ForteBio, Sartorius).

### 4.19. Flow Cytometry Binding Analysis

Binding of each mAb to NFS-60 (constitutively expressing mβc) and M1 (devoid of mβc) cells was assessed to determine specificity toward the target receptor. Approximately 2.5 × 10^5^ cells were used per sample, plated in 96-well round bottom plates, washed, and resuspended in 25 µL PBS supplemented with 10% goat serum. Each antibody was added at a final concentration of 200 µg/mL in a total volume of 50 µL. A commercial mIgG2a isotype control antibody was included as a negative control. Cells were incubated with each mAb for 1 h at 4 °C, then washed 2 times, and resuspended in 50 µL of a PE-conjugated secondary goat anti-mouse IgG antibody at a final dilution of 1:50. Cells were incubated at 4 °C for 1 h in the dark, washed 3 times, then resuspended in 200 µL PBS supplemented with 2% FBS. Expression levels of mIL-3Rα, mβc, and mβ_IL-3_ were assessed by staining cells with PE-conjugated rat anti-mouse CD123 (mIL3Rα) and CD131 (mβc) antibodies and an Alexa Fluor 488 conjugated rat anti-mouse mβ_IL-3_ antibody, all at a final concentration of 2 μg/mL. Samples were measured on a CytoFLEX flow cytometer, and data were processed using FlowJo v10.6.2 (FlowJo LLC).

### 4.20. Intracellular Staining for Phosphorylated STAT5 (pSTAT5)

Detection of pSTAT5 as an indicator of IL-3 mediated signaling was performed on NFS-60 cells to determine the inhibitory potential of candidate mAbs. NFS-60 cells were cytokine starved overnight and then starved of FBS 1 h prior to the assay. Cells were plated at 4 × 10^5^ cells per well in a 96-well round bottom plate and then pelleted by centrifugation. The pellet was resuspended in pre-warmed media containing mAbs at various dilutions, followed by incubation for 30 min at 37 °C. Cells were stimulated with 2 ng/mL of mIL-3 in RPMI medium for 20 min (or with RPMI medium only for non-stimulated controls), then washed with PBS, fixed with 4% PFA at 4 °C for 20 min, washed, then permeabilized using 90% methanol at 4 °C for 30 min. Cells were resuspended in a PE mouse anti-pSTAT5 (pY694) antibody diluted 1:10 prior to incubation in the dark at 4 °C for 1 h. Cells were prepared for flow cytometry analysis as described above.

### 4.21. Statistics

All statistical analyses and figures were generated using GraphPad Prism Software v. 9 (GraphPad Software). For the cell signaling assay, replicates of the median fluorescence intensity (MFI) readings from flow cytometry were used to perform a One-Way ANOVA with a Tukey’s multiple comparisons test to determine significance.

## Data Availability

All available data were published and are further available from the authors.

## Supplementary Materials

The following are available online at https://www.mdpi.com/article/10.3390/ijms23158470/s1.
