# Supplementary material for: Evaluation of Phage Display Biopanning Strategies for the Selection of Anti-Cell Surface Receptor Antibodies
Source: Int J Mol Sci. 2022 Jul 30;23(15):8470. doi: 10.3390/ijms23158470 (PMC9369378; doi:10.3390/ijms23158470)
Supplement: Supplementary file 1 [file ijms-23-08470-s001.zip › Supplementary File S1--Figures S1-S14 Tables S1-S7.pdf]

Supplementary Information

Evaluation of phage display biopanning strategies for the selection of anti-cell surface receptor antibodies

Nadya Panagides[1], Lucia F Zacchi[1], Mitchell J De Souza[2], Rodrigo AV Morales[2], Alexander Karnowski[2], Mark T Liddament[2], Catherine M Owczarek[2], Stephen M Mahler[1], Con Panousis[2], Martina L Jones[1] and Christian Fercher[1*]

[1] ARC Training Centre for Biopharmaceutical Innovation, Australian Institute for Bioengineering and Nanotechnology (AIBN), University of Queensland, Brisbane, Australia. n.panagides@uq.edu.au (NP); l.zacchi@uq.edu.au (LFZ); s.mahler@eng.uq.edu.au (SMM); martina.jones@uq.edu.au (MLJ); c.fercher@uq.edu.au (CF)

[2] Research and Development, CSL Limited; Bio21 Molecular Science and Biotechnology Institute, Parkville Victoria, 3010, Australia. mitchell.desouza@csl.com.au (MJDS); rodrigo.morales@csl.com.au (RAVM); alexander.karnowski@csl.com.au (AK); mark.liddament@csl.com.au (MTL); catherine.owczarek@csl.com.au (CMO); kosta.panousis@csl.com.au (CP)

***** Correspondence: c.fercher@uq.edu.au Tel: +61 7 334 64280

Supplementary Tables

Supplemental Table S1. Phage input and output titer for each selection method and biopanning round. Prefix s – surface-tethered recombinant ECD (STRE); c – cell-based antigen (CBA).

Code	Sample Description	Input Titer [cfu]	Output Titer [cfu]
**JM**	Jones-Mahler scFv phage library	1.0×10^{13}	-
**sR1**	STRE biopanning round 1	8.0×10^{12}	6×10^{7}
**sR2**	STRE biopanning round 2	6.3×10^{12}	1×10^{6}
**sR3**	STRE biopanning round 3	8.0×10^{12}	2×10^{8}
**cR1**	CBA biopanning round 1	5.5×10^{12}	5×10^{4}
**cR2**	CBA biopanning round 2	2.8×10^{12}	1×10^{6}
**cR3**	CBA biopanning round 3	6.0×10^{12}	1×10^{9}

Supplemental Table S2. Positive binders isolated from round 3 phage pools including their CDR-H3 sequences and length. Clones further pursued in this work are underlined. Prefix s – STRE; c – CBA.

	Positive phage clones	CDR-H3
**1**	sF5	-----AKVGTI--FGVSYYYMDV--- 16
**2**	sA10,sG2,sG6<br>cA8,cA12,cB3,cC8,cD1,cD7,cE6,cF4	----------A--RKATWDGMDV--- 11<br>----------A--RKATWDGMDV--- 11
**3**	sC3	---ARDIRIQL--WPNYYYGMDV--- 18
**4**	sB5,sF11	---------------ARKYHMDV--- 8
**5**	sC9	---------AK--ELAYYYGMDV--- 12
**6**	sA5,sC7,sD7,sD9,sE8,sF7,sG11<br>cA3,cA10,cB5,cD3,cD6,cE4,cF1,cF7,cF12,cG3,cG4,cG7,cG12,cH6,cH7,cH10	---ARGNY-GG--NGGATNWFDL--- 17<br>---ARGNY-GG--NGGATNWFDL--- 17
**7**	cB8,cC4	----AREY-RS----GWYYGFDI--- 14
**8**	sE2	-------A-RE----VDYYGLDV--- 11
**9**	sA12,sB6,sC6,sC12	---ARDGL-GG----RAFNGMDV--- 15
**10**	sA2, sD11	---AR-----------EGGGDPPGDY 12
**11**	sG8	---AR-----------NGGGMDV--- 9
**12**	sD1,sE7,sG4	---AF-----------QAYGMDV--- 9
**13**	cB10	---AR-----------DRYGMDV--- 9
**14**	sF2	---ARDREWELLYY------------ 11
**15**	sB10	---ARDTNWGLDF------------- 10
**16**	sH8,sD6<br>cA1,cA9,cB2,cC3,cC7,cC10,cD4,cE2,cF11	---ARRGIFGVENLDA----FDI--- 16<br>---ARRGIFGVENLDA----FDI--- 16
**17**	sA8,sB11,sE5,sE6,sF10,sE9,sF3<br>cD2	---ARDQDTGVL-MDA----FDI--- 15<br>---ARDQDTGVL-MDA----FDI--- 15
**18**	sF9<br>cB7	ARRGRSTIFGVV-NDA----FDI--- 18<br>ARRGRSTIFGVV-NDA----FDI--- 18
**19**	sB2	---ARDLGLYCSGGSCYENGMDV--- 20
**20**	sC10	---ARELGFYASGSGT---------- 13
**21**	sD5	---ARDLGWSRG--GA----FDI--- 14
**22**	sD10,sH12	--ARRGYDF-SD--DA----FD---- 13
**23**	sG10	--ARVGSGW-RI--DA----FDI--- 14
**24**	cG8	-ARRIWSGY-FD--DA----FDI--- 15
**25**	sH10	---ARDS----GDNWNYEGDY----- 14
**26**	sF6,sG3<br>cC12,cH8	----------AREGWS---YFDL--- 10<br>----------AREGWS---YFDL--- 10
**27**	sC1	----------AREGSMMGDAFDI--- 13
**28**	sG12	-----------SSSSPEGDAFDI--- 12
**29**	cA5	---AKDTYHGKYSSSWYLDAFDI--- 20
**30**	cE5	----------TTEEGSSGGAFHI--- 13
**31**	sD12<br>cB1,cG6	---AREEFAVVKWGVQQRRAFDI--- 20<br>---AREEFAVVKWGVQQRRAFDI--- 20
**32**	sF4	-------------ARGSFLDY----- 8
**33**	sC5,sD2	-------------ARGSRGAFDI--- 10

Supplemental Table S3. Melting temperature (Tm) measurements of anti-mβc mAbs determined using differential scanning fluorometry (DSF). Clones were ranked from lowest to highest Tm.

Clone	T_m [°C]	Clone	T_m [°C]
sF5	50	sD5	64
sB5	51	sC12	65
sE2	57	cG8	66
F10	57	sB2	66
sA2	58	sC9	66
sG12	58	sC3	66
sG11	59	sF2	67
sC1	60	cB10	67
sF4	60	cC4	67
sG3	61	sA10	67
cE5	61	sE7	67
sG10	61	sG8	68
sH8	62	cA5	68
sC10	64	sC5	69
sB10	64	sH12	70

Supplemental Table S3. Dissociation (k_d) kinetics of mAbs that gave sensorgrams that could be reliably fitted with a 1:1 stoichiometric binding model. Clones were ranked from fastest to slowest dissociation kinetics.

Rank	Clone	k_d [1/s]
**1**	**sF5**	$3.91x10^{-4}$
**2**	**sE7**	$9.77x10^{-4}$
**3**	**sC3**	$1.64x10^{-3}$
**4**	**H8**	$1.73x10^{-3}$
**5**	**sF2**	$6.57x10^{-3}$
**6**	**G3**	$7.99x10^{-3}$
**7**	**sD5**	$2.38x10^{-2}$
**8**	**sC1**	$2.98x10^{-2}$
**9**	**F10**	$3.23x10^{-2}$
**10**	**sG10**	$7.78x10^{-2}$
**11**	**G11**	$1.34x10^{1}$

Supplemental Table S5. *Escherichia coli* bacterial strains and corresponding phenotypes used in this work.

Strain	Genotype
**XL1-Blue (Stratagene)**	*recA1 endA1 gyrA96 thi-1 hsdR17 supE44 relA1 lac [F´ proAB lacIq ZΔM15 Tn10 (Tet^R)]*
**Top10 (Invitrogen)**	*F- mcrA Δ(mrr-hsdRMS-mcrBC) φ80lacZΔM15 ΔlacX74 nupG recA1 araD139 Δ(ara-leu)7697 galE15 galK16 rpsL(Str^R) endA1 λ-*
**α-Select (BioLine)**	*F - deoR endA1 recA1 relA1 gyrA96 hsdR17(rk -, mk +) supE44 thi-1 phoA Δ(lacZYA argF)U169 Φ80lacZΔM15λ*
**Shuffle cells (New England BioLabs)**	*fhuA2 [lon] ompT ahpC gal λatt::pNEB3-r1-cDsbC (Spec^R, lacIq) ΔtrxB sulA11 R(mcr-73::miniTn10--Tet^S)2 [dcm] R(zgb-210::Tn10 --Tet^S) endA1 Δgor Δ(mcrC-mrr)114::IS10*

Supplemental Table S6. PCR amplification and sequencing primers for scFv containing phagemids.

Primer Name	Primer Sequence (5'– 3')
**pPhNBF2_PCR_Forward primer**	TTGTGTGGAATTGTGAGCGG
**pPhNBF2_PCR_Reverse primer**	CCTTCATAATTTGCATAGCGATCCAGGG
**pPhNBF2_Sequencing_Forward primer**	CAGGAAACAGCTATGACC
**pPhNBF2_Sequencing_Reverse primer**	TTTCAACGGTCTATGCCGGCACC

Supplemental Table S7. Primer sequences for amplification and sequencing of anti-mβc variable chains for insertion into mouse IgG2a constant heavy and light chain backbones.

mAbXpress primer for scFv variable regions			
Each variable primer sequence is preceded by the following sequences			
**HEAVY CHAIN (5'-3')**		**LIGHT CHAIN (5'-3')**	
**Forward**	**Reverse**	**Forward**	**Reverse**
CAGGTGTCCACTCCGC	GCGGAGGACACGGTGAG	CCGGCGTGCACTCCGAG	GCCTTAGGCTGGCCGAG (λ)<br>GGCCACGGTCCGCTTGAG (κ)

**CLONE** (λ or κ)	**HEAVY CHAIN (5'-3')**		**LIGHT CHAIN (5'-3')**	
	**Forward**	**Reverse**	**Forward**	**Reverse**
**A10** (λ)	**mAbX_mVhF8**<br>GTGCAGCTACAACAG	**mAbX_VhR1**<br>GGGCCAAGGGACCACG	**mAbX_VLF15**<br>TATGAGCTGACGCAGCC	**mAbX_VLR4**<br>GGACCAAGCTGACCGTC
**D12** (λ)	**mAbX_mVhF14**<br>ATGCAGCTGGTGCAG	**mAbX_VhR6**<br>GGCCAAGGGACAATG	**mAbX_VLF18**<br>ATTGTGTTGACTCAGTC	**mAbX_VLR7**<br>GGGACAAAGGTGGATATTAAAC
**F9** (λ)	**mAbX_mVhF6**<br>GTKCAGCTGGTRCAG	**mAbX_VhR6**<br>GGCCAAGGGACAATG	**mAbX_VLF3**<br>TCTGTGTTGACGCAGCCG	**mAbX_VLR4**<br>GGACCAAGCTGACCGTC
**F10** (λ)	**mAbX_mVhF6**<br>GTKCAGCTGGTRCAG	**mAbX_VhR6**<br>GGCCAAGGGACAATG	**mAbX_VLF15**<br>TATGAGCTGACGCAGCC	**mAbX_VLR4**<br>GGACCAAGCTGACCGTC
**G3** (λ)	**mAbX_mVhF15**<br>GTGCAGCTGTTGCAG	**mAbX_VhR5**<br>GGGGCCGTGGCACCCTG	**mAbX_VLF16**<br>TATGAGCTGACACAGTC	**mAbX_VLR4**<br>GGACCAAGCTGACCGTC
**G11** (λ)	**mAbX_mVhF5**<br>GTGCAGCTGGTGGAG	**mAbX_VhR5**<br>GGGGCCGTGGCACCCTG	**mAbX_VLF16**<br>TATGAGCTGACACAGTC	**mAbX_VLR4**<br>GGACCAAGCTGACCGTC
**H8** (λ)	**mAbX_mVhF15**<br>GTGCAGCTGTTGCAG	**mAbX_VhR6**<br>GGCCAAGGGACAATG	**mAbX_VLF2**<br>TCTGCTCTGACTCAGCC	**mAbX_VLR4**<br>GGACCAAGCTGACCGTC
**sA2** (λ)	**mAbX_mVhF5**<br>GTGCAGCTGGTGGAG	**mAbX_VhR2**<br>GGCCAGGGAACCCTG	**mAbX_VLF15**<br>TATGAGCTGACGCAGCC	**mAbX_VLR4**<br>GGACCAAGCTGACCGTC
**sB2** (λ)	**mAbX_mVhF5**<br>GTGCAGCTGGTGGAG	**mAbX_VhR1**<br>GGGCCAAGGGACCACG	**mAbX_VLF17**<br>TCTGTGTTGACTCAGCC	**mAbX_VLR4**<br>GGACCAAGCTGACCGTC
**sB5** (λ)	**mAbX_mVhF5**<br>GTGCAGCTGGTGGAG	**mAbX_VhR7**<br>GGCAAAGGGACCACG	**mAbX_VLF15**<br>TATGAGCTGACGCAGCC	**mAbX_VLR4**<br>GGACCAAGCTGACCGTC
**sB10** (λ)	**mAbX_mVhF14**<br>ATGCAGCTGGTGCAG	**mAbX_VhR2**<br>GGCCAGGGAACCCTG	**mAbX_VLF15**<br>TATGAGCTGACGCAGCC	**mAbX_VLR4**<br>GGACCAAGCTGACCGTC
**sC1** (λ)	**mAbX_mVhF6**<br>GTKCAGCTGGTRCAG	**mAbX_VhR6**<br>GGCCAAGGGACAATG	**mAbX_VLF16**<br>TATGAGCTGACACAGTC	**mAbX_VLR4**<br>GGACCAAGCTGACCGTC
**sC3** (κ)	**mAbX_mVhF6**<br>GTKCAGCTGGTRCAG	**mAbX_VhR1**<br>GGGCCAAGGGACCACG	**mAbX_VkF11**<br>ATTGTGATGACACAG	**mAbX_VkR1**<br>TTCCAACTTTGTCCC
**sC5** (κ)	**mAbX_mVhF7**<br>GTGCARCTKCAGCAG	**mAbX_VhR6**<br>GGCCAAGGGACAATG	**mAbX_VkF12**<br>ATCTGGATGACCCAG	**mAbX_VkR7**<br>GGGACCAAACTGGAA
**sC9** (λ)	**mAbX_mVhF6**<br>GTKCAGCTGGTRCAG	**mAbX_VhR1**<br>GGGCCAAGGGACCACG	**mAbX_VLF15**<br>TATGAGCTGACGCAGCC	**mAbX_VLR4**<br>GGACCAAGCTGACCGTC
**sC10** (λ)	**mAbX_mVhF6**<br>GTKCAGCTGGTRCAG	**mAbX_VhR2**<br>GGCCAGGGAACCCTG	**mAbX_VLF3**<br>TCTGTGTTGACGCAGCCG	**mAbX_VLR4**<br>GGACCAAGCTGACCGTC
**sC12** (λ)	**mAbX_mVhF16**<br>GTCCAGCTKGTACAG	**mAbX_VhR8**<br>CAGGGTGCCGTGGCC	**mAbX_VLF15**<br>TATGAGCTGACGCAGCC	**mAbX_VLR4**<br>GGACCAAGCTGACCGTC
**sD5** (λ)	**mAbX_mVhF15**<br>GTGCAGCTGTTGCAG	**mAbX_VhR6**<br>GGCCAAGGGACAATG	**mAbX_VLF16**<br>TATGAGCTGACACAGTC	**mAbX_VLR4**<br>GGACCAAGCTGACCGTC
**sE2** (λ)	**mAbX_mVhF5**<br>GTGCAGCTGGTGGAG	**mAbX_VhR1**<br>GGGCCAAGGGACCACG	**mAbX_VLF16**<br>TATGAGCTGACACAGTC	**mAbX_VLR4**<br>GGACCAAGCTGACCGTC
**sE7** (λ)	**mAbX_mVhF6**<br>TKCAGCTGGTRCAG	**mAbX_VhR1**<br>GGGCCAAGGGACCACG	**mAbX_VLF2**<br>TCTGCTCTGACTCAGCC	**mAbX_VLR4**<br>GGACCAAGCTGACCGTC
**sF2** (λ)	**mAbX_mVhF6**<br>GTKCAGCTGGTRCAG	**mAbX_VhR2**<br>GGCCAGGGAACCCTG	**mAbX_VLF2**<br>TCTGCTCTGACTCAGCC	**mAbX_VLR4**<br>GGACCAAGCTGACCGTC

**sF4** (λ)	**mAbX_mVhF6**<br>GTKCAGCTGGTRCAG	**mAbX_VhR2**<br>GGCCAGGGAACCCTG	**mAbX_VLF2**<br>TCTGCTCTGACTCAGCC	**mAbX_VLR4**<br>GGACCAAGCTGACCGTC
**sF5** (λ)	**mAbX_mVhF5**<br>GTGCAGCTGGTGGAG	**mAbX_VhR1**<br>GGGCCAAGGGACCACG	**mAbX_VLF15**<br>TATGAGCTGACGCAGCC	**mAbX_VLR4**<br>GGACCAAGCTGACCGTC
**sG8** (λ)	**mAbX_mVhF6**<br>GTKCAGCTGGTRCAG	**mAbX_VhR1**<br>GGGCCAAGGGACCACG	**mAbX_VLF15**<br>TATGAGCTGACGCAGCC	**mAbX_VLR4**<br>GGACCAAGCTGACCGTC
**sG10** (λ)	**mAbX_mVhF5**<br>GTGCAGCTGGTGGAG	**mAbX_VhR6**<br>GGCCAAGGGACAATG	**mAbX_VLF16**<br>TATGAGCTGACACAGTC	**mAbX_VLR4**<br>GGACCAAGCTGACCGTC
**sG12** (λ)	**mAbX_mVhF5**<br>GTGCAGCTGGTGGAG	**mAbX_VhR6**<br>GGCCAAGGGACAATG	**mAbX_VLF16**<br>TATGAGCTGACACAGTC	**mAbX_VLR4**<br>GGACCAAGCTGACCGTC
**sH10** (λ)	**mAbX_mVhF3**<br>GTGCAGCTGCAGGAG	**mAbX_VhR2**<br>GGCCAGGGAACCCTG	**mAbX_VLF3**<br>TCTGTGTTGACGCAGCCG	**mAbX_VLR4**<br>GGACCAAGCTGACCGTC
**sH12** (λ)	**mAbX_mVhF16**<br>GTCCAGCTKGTACAG	**mAbX_VhR6**<br>GGCCAAGGGACAATG	**mAbX_VLF15**<br>TATGAGCTGACGCAGCC	**mAbX_VLR4**<br>GGACCAAGCTGACCGTC
**cA5** (λ)	**mAbX_mVhF6**<br>GTKCAGCTGGTRCAG	**mAbX_VhR6**<br>GGCCAAGGGACAATG	**mAbX_VLF17**<br>TCTGTGTTGACTCAGCC	**mAbX_VLR4**<br>GGACCAAGCTGACCGTC
**cB10** (λ)	**mAbX_mVhF5**<br>TGCAGCTGGTGGAG	**mAbX_VhR7**<br>GGGCAAAGGGACCACG	**mAbX_VLF15**<br>TATGAGCTGACGCAGCC	**mAbX_VLR4**<br>GGACCAAGCTGACCGTC
**cC4** (λ)	**mAbX_mVhF14**<br>ATGCAGCTGGTGCAG	**mAbX_VhR6**<br>GGCCAAGGGACAATG	**mAbX_VLF15**<br>TATGAGCTGACGCAGCC	**mAbX_VLR4**<br>GGACCAAGCTGACCGTC
**cE5** (λ)	**mAbX_mVhF5**<br>GTGCAGCTGGTGGAG	**mAbX_VhR6**<br>GGCCAAGGGACAATG	**mAbX_VLF16**<br>TATGAGCTGACACAGTC	**mAbX_VLR4**<br>GGACCAAGCTGACCGTC
**cG8** (λ)	**mAbX_mVhF14**<br>ATGCAGCTGGTGCAG	**mAbX_VhR6**<br>GGCCAAGGGACAATG	**mAbX_VLF17**<br>TCTGTGTTGACTCAGCC	**mAbX_VLR4**<br>GGACCAAGCTGACCGTC
**A10** (λ)	**mAbX_mVhF8**<br>GTGCAGCTACAACAG	**mAbX_VhR1**<br>GGGCCAAGGGACCACG	**mAbX_VLF15**<br>TATGAGCTGACGCAGCC	**mAbX_VLR4**<br>GGACCAAGCTGACCGTC
**D12** (λ)	**mAbX_mVhF14**<br>ATGCAGCTGGTGCAG	**mAbX_VhR6**<br>GGCCAAGGGACAATG	**mAbX_VLF18**<br>ATTGTGTTGACTCAGTC	**mAbX_VLR7**<br>GGGACAAAGGTGGATATTAAAC
**F9** (λ)	**mAbX_mVhF6**<br>GTKCAGCTGGTRCAG	**mAbX_VhR6**<br>GGCCAAGGGACAATG	**mAbX_VLF3**<br>TCTGTGTTGACGCAGCCG	**mAbX_VLR4**<br>GGACCAAGCTGACCGTC
**F10** (λ)	**mAbX_mVhF6**<br>GTKCAGCTGGTRCAG	**mAbX_VhR6**<br>GGCCAAGGGACAATG	**mAbX_VLF15**<br>TATGAGCTGACGCAGCC	**mAbX_VLR4**<br>GGACCAAGCTGACCGTC

mAbXpress sequencing primers

**UNIVERSAL**	**HEAVY CHAIN (5'-3')**	**LIGHT CHAIN (5'-3')**	
**Forward**	**Reverse**	**Kappa Reverse**	**Lambda Reverse**
**mAbX_SeqF**<br>TAACACCGCCCCGGTTTTCC	**mAbX_Seq-mG2a-R**<br>GCCTTTCACCAGGCAGCC	**mAbX_Seq-mKapR**<br>GTTCAGGAAGCAGACCACGG	**mAbX_Seq-mLamR**<br>GGTGCACACGAGGGTGGCC

Supplementary Figures

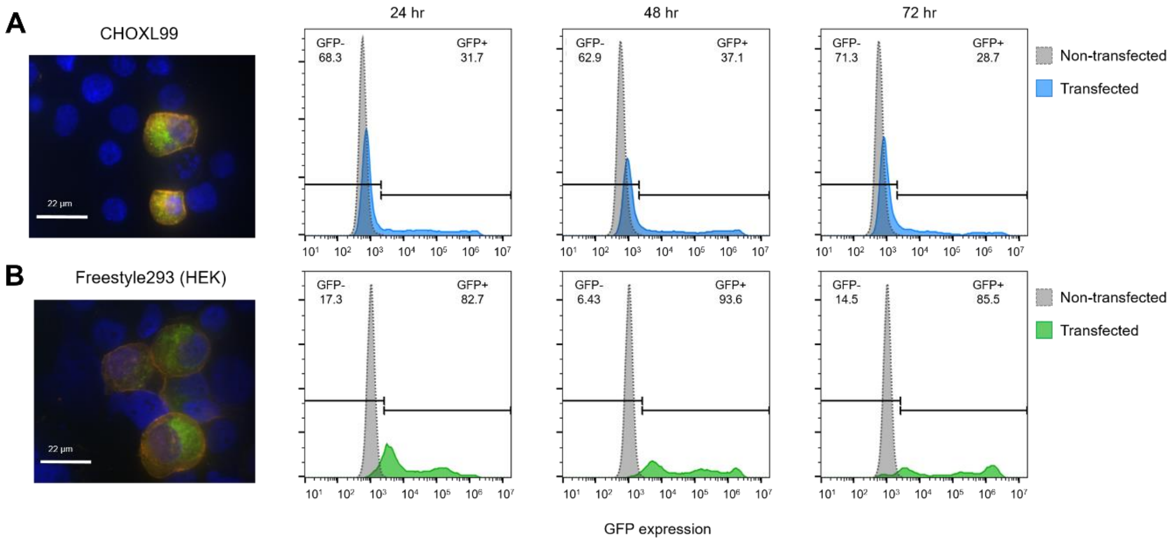

Supplemental Figure S1. Transfection efficiency analysis of CHO-XL99 (A) and Freestyle™ 293-F (HEK) (B) cells via fluorescence microscopy (GFP – green, Hoechst 33342 nuclear stain – blue) and flow cytometry. Cells were transfected with a mammalian expression plasmid harboring a mβc-GFP fusion construct and GFP fluorescence was assessed 24, 48 and 72 h post-transfection to determine the optimum time point to commence CBA biopanning.

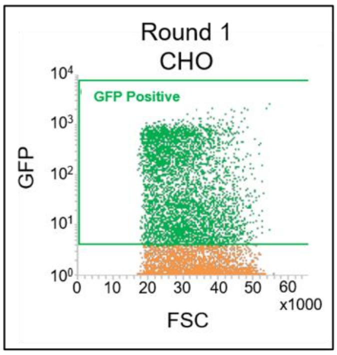

Populations	Events	% Total
All Events	50,000	100.00%
Cells	28,160	56.32%
Single Cells	27,768	55.54%
GFP Positive	4,826	9.65%

Event Count	Sort Count
16,813,890	1,108,386

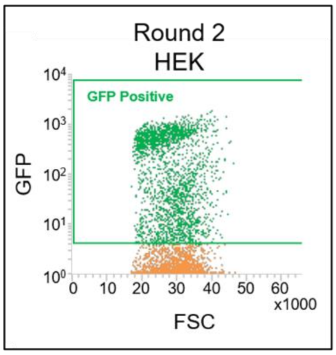

Populations	Events	% Total
All Events	30,976	100.00%
Cells	5,265	17.00%
Single Cells	5,202	16.79%
GFP Positive	2,392	7.72%

Event Count	Sort Count
10,605,956	687,703

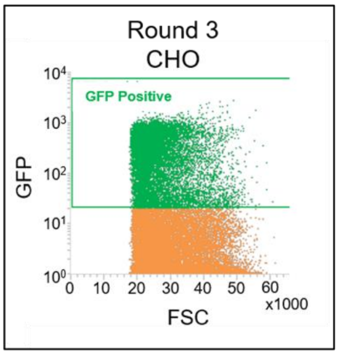

Populations	Events	% Total
All Events	202,455	100.00%
Cells	117,206	57.89%
Single Cells	114,702	56.66%
GFP Positive	12,868	6.36%

Event Count	Sort Count
23,655,176	1,014,361

Supplemental Figure S2. FACS reports and gate settings for the isolation of GFP-positive cells in CBA biopanning. Dot plots (left) show GFP expression levels vs forward scatter (FSC) indicating gates used for positive cell selection for each biopanning round. The gate threshold for round 3 was raised to increase stringency. Corresponding hierarchical population statistics and final event and sort counts are shown on the right. The viability of CHO-XL99 cells in round 1 and 3 was approximately 57% with $>1x10^6$ cells recovered while Freestyle™ 293-F cell viability was lower at 17% with $<0.7x10^6$ cells recovered.

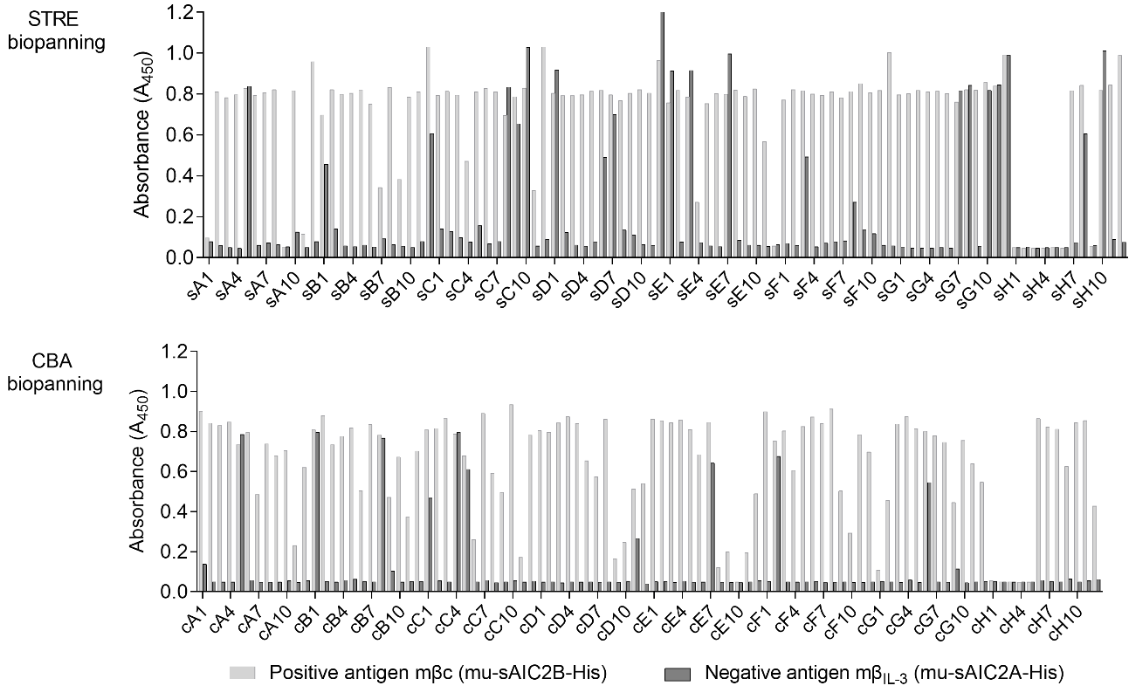

Supplemental Figure S3. Monoclonal phage ELISA of all clones isolated from round 3 phage pools generated during STRE (A) and CBA (B) biopanning. The positive (mβc) and negative ($m\beta_{IL\text{-}3}$) antigens were coated on ELISA plates at 3 µg/mL. Phage particles were detected using an anti-M13 HRP-conjugated antibody. Wells H1-H5 served as negative controls and did not contain phage particles.

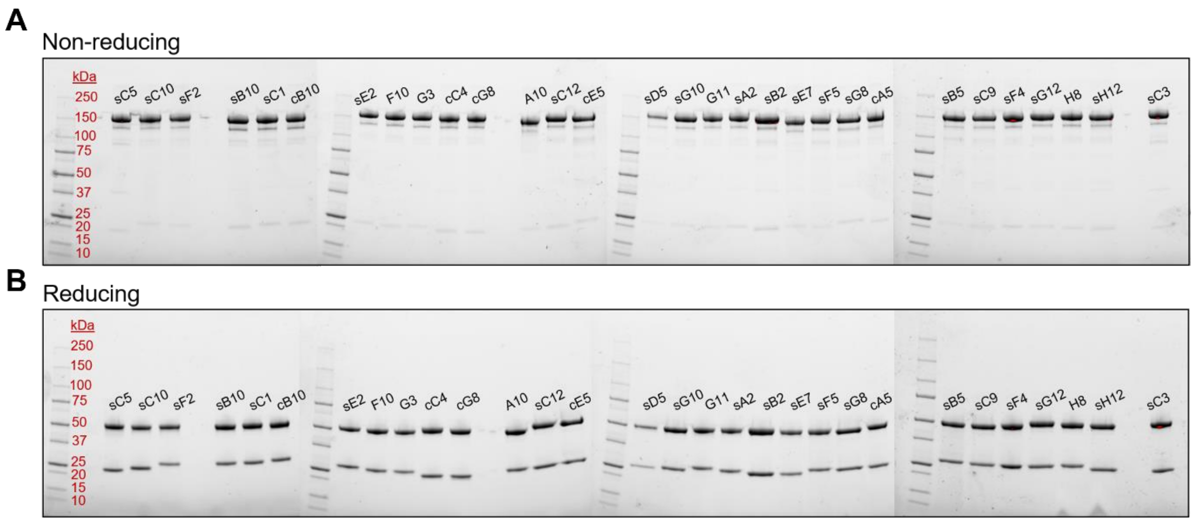

Supplemental Figure S4. SDS-PAGE analysis of 30 purified anti-mβc mAbs. A total of 5 µg of each antibody was loaded and analyzed under non-reducing (A) and reducing (B) conditions. A major band at around 150 kDa in the non-reducing gels indicates correct assembly of heavy and light chains.

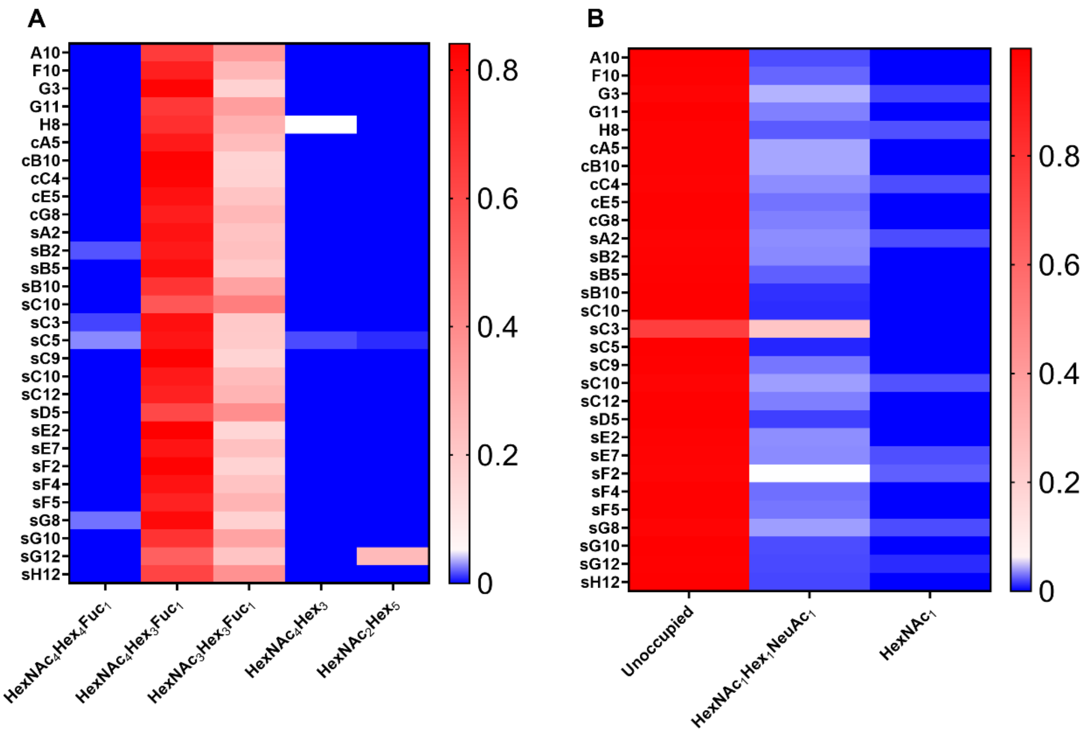

Supplemental Figure S5. Heatmaps depicting the relative abundance of glycoforms identified for each anti-mβc mAb by mass spectrometry. (A) *N*-linked glycans found in the peptide $E_{319}DYN_{322}STLR_{326}$ of the mIgG2a Fc region. The sequon was 100% occupied, as expected. (B) *O*-linked glycosylation in peptide $GPT_{247}IKPCPPCL$ of the hinge region is significantly less abundant as the peptide was predominantly unoccupied. The bar indicates the abundance of each glycoform relative to the sum of the abundances of all forms of that glycopeptide.

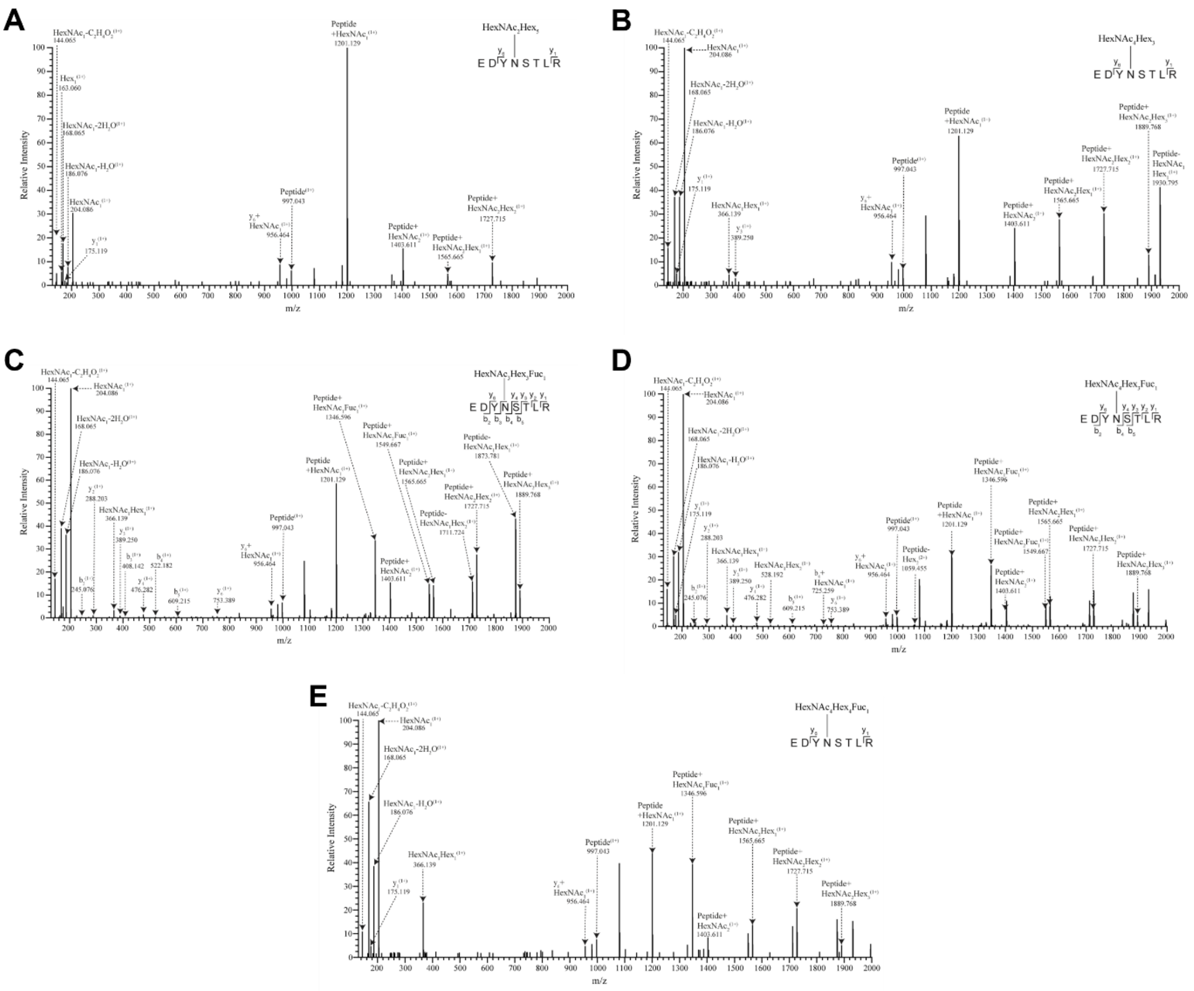

Supplemental Figure S6. Representative higher energy collisional dissociation (HCD) spectra of glycopeptides featuring the *N*-linked glycosylation site at Asn322 in the Fc region of anti-mβc mAbs. (A) $HexNAc_2Hex_5$ and (B) $HexNAc_4Hex_3$ derived from clone sC5, (C) $HexNAc_3Hex_3Fuc_1$ derived from G11, (D) $HexNAc_4Hex_3Fuc_1$ derived from clone cB10 and (E) $HexNAc_4Hex_4Fuc_1$ derived from sC5. For clarity, not all fragment ions in the spectra were labelled.

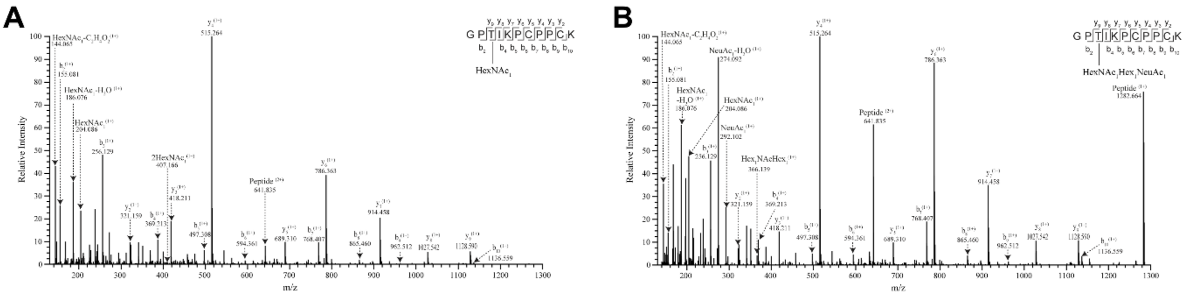

Supplemental Figure S7. Representative HCD spectra of glycopeptides containing the *O*-linked glycosylation site Thr247 in the hinge region of anti-mβc mAbs. (A) HexNAc derived from clone sC10 and (B) $HexNAc_1Hex_1NeuAc_1$ derived from clone sC5. For clarity, not all fragment ions in the spectra were labelled.

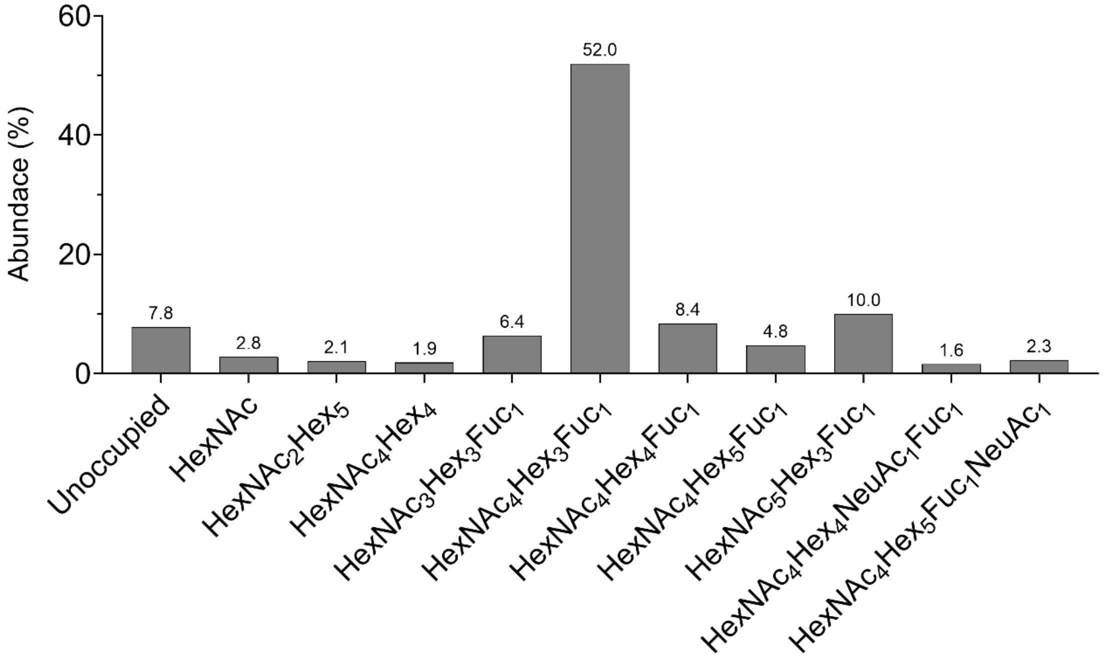

Supplemental Figure S8. Relative abundance of individual glycoforms identified in the variable heavy chain region of mAb sC12. Both *N*- and *O*-linked glycosylation sites were found to contribute to the partial occupancy of the peptide T$_{43}$SGYN$_{47}$FT$_{49}$SYAMHWVR$_{57}$.

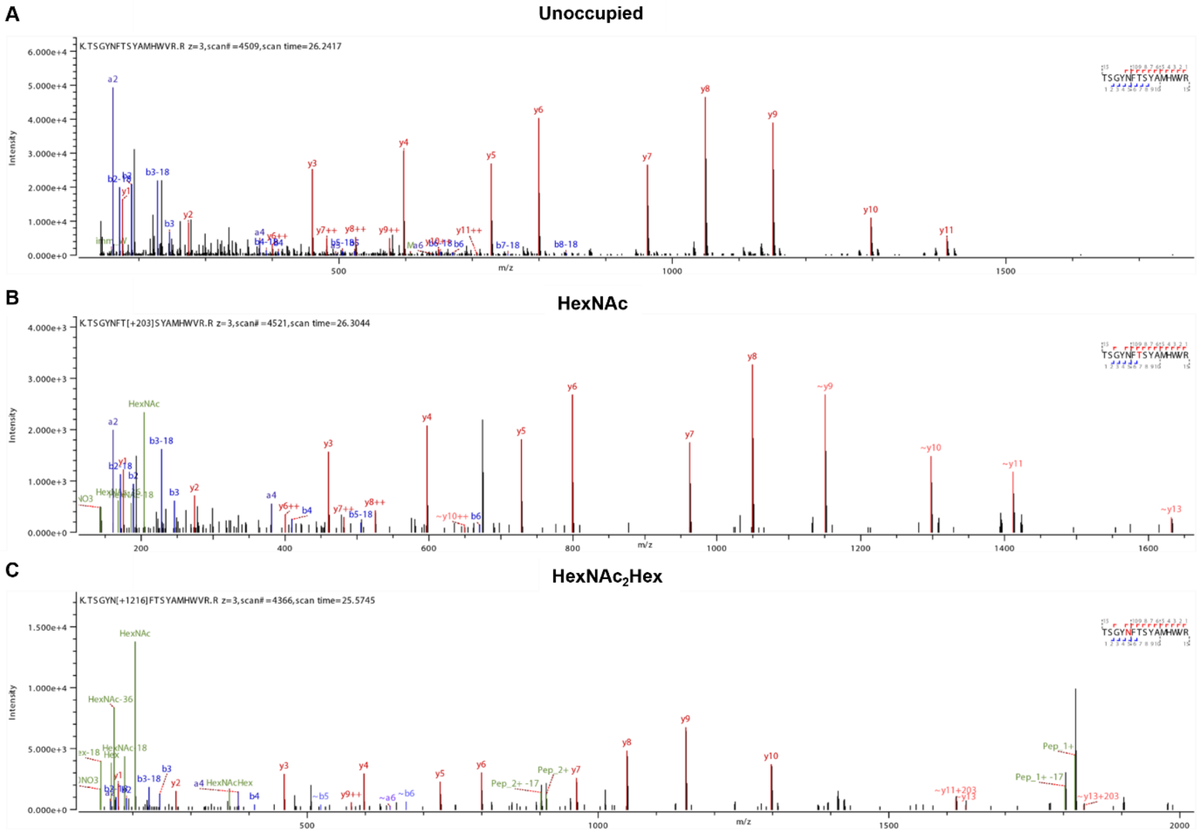

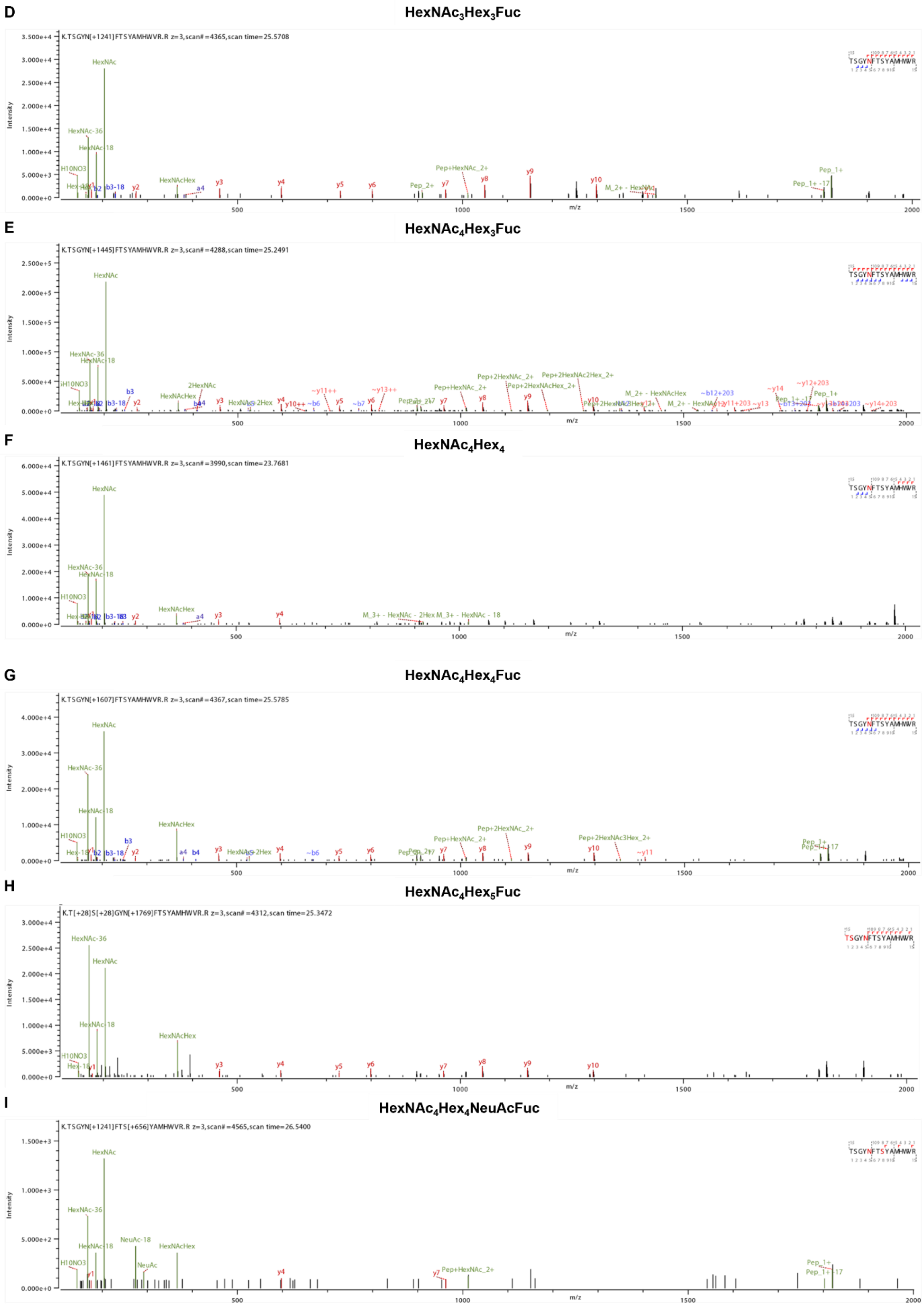
D
$HexNAc_3Hex_3Fuc$
K.TSGYN[+1241]FTSYAMHWVR.R z=3,scan#=4365,scan time=25.5708
E
$HexNAc_4Hex_3Fuc$
K.TSGYN[+1445]FTSYAMHWVR.R z=3,scan#=4288,scan time=25.2491
F
$HexNAc_4Hex_4$
K.TSGYN[+1461]FTSYAMHWVR.R z=3,scan#=3990,scan time=23.7681
G
$HexNAc_4Hex_4Fuc$
K.TSGYN[+1607]FTSYAMHWVR.R z=3,scan#=4367,scan time=25.5785
H
$HexNAc_4Hex_5Fuc$
K.T[+28]S[+28]GYN[+1769]FTSYAMHWVR.R z=3,scan#=4312,scan time=25.3472
I
$HexNAc_4Hex_4NeuAcFuc$
K.TSGYN[+1241]FTS[+656]YAMHWVR.R z=3,scan#=4565,scan time=26.5400
Intensity
m/z

J

HexNAc$_4$Hex$_5$FucNeuAc

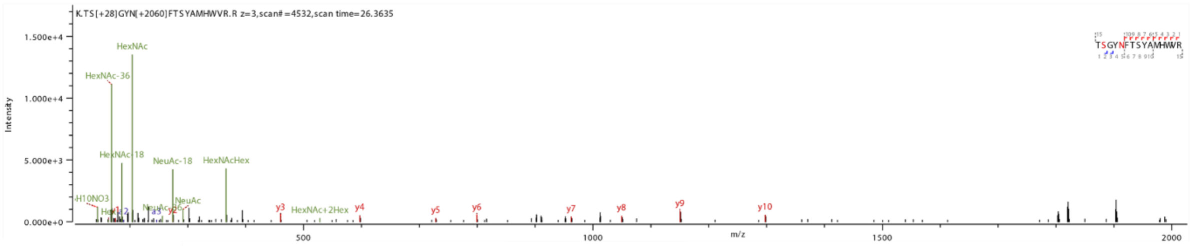

K

HexNAc$_5$Hex$_3$Fuc

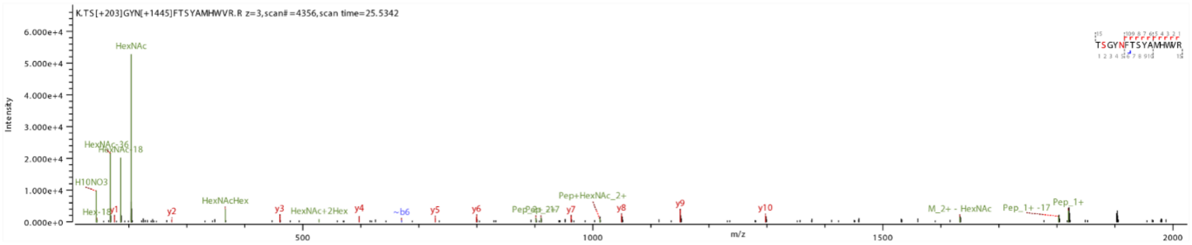

Supplemental Figure S9. HCD mass spectra of glycopeptides containing the *N*-linked glycosylation site Asn47 and the *O*-linked glycosylation site Thr49 in the variable region of mAb sC12. (A) Unoccupied, (B) *O*-linked glycan HexNAc, (C-K) *N*-linked glycans HexNAc$_2$Hex$_1$, HexNAc$_3$Hex$_3$Fuc$_1$, HexNAc$_4$Hex$_3$Fuc$_1$, HexNAc$_4$Hex$_4$, HexNAc$_4$Hex$_4$Fuc$_1$, HexNAc$_4$Hex$_5$Fuc$_1$, HexNAc$_4$Hex$_4$NeuAc$_1$Fuc$_1$, HexNAc$_4$Hex$_5$Fuc$_1$NeuAc$_1$ and HexNAc$_5$Hex$_3$Fuc$_1$.

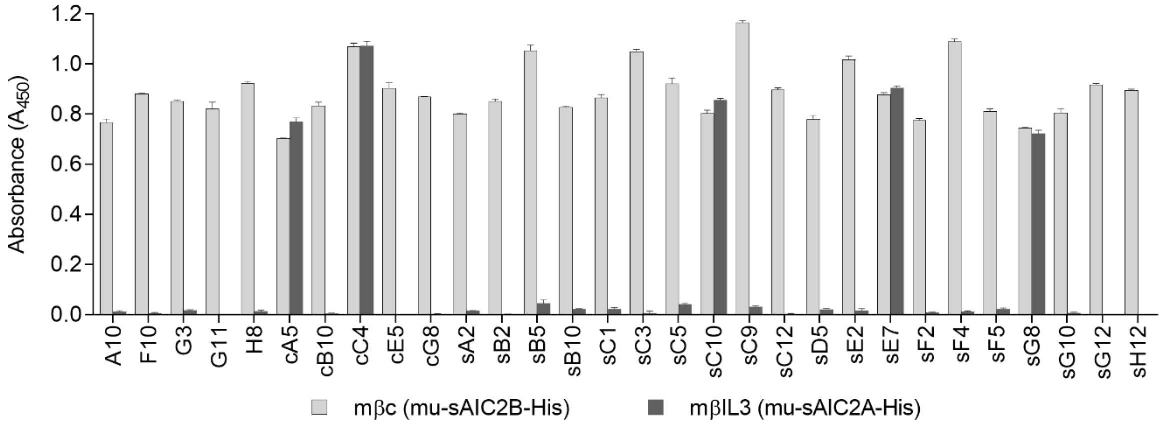

Supplemental Figure S10. ELISA of purified anti-mβc mAbs against soluble recombinant mβc and mβ$_{IL-3}$. Binding was assessed in triplicate using 10 µg/mL of mAbs against 5 µg/mL antigens coated on the plate. Data is shown as mean ± SEM.

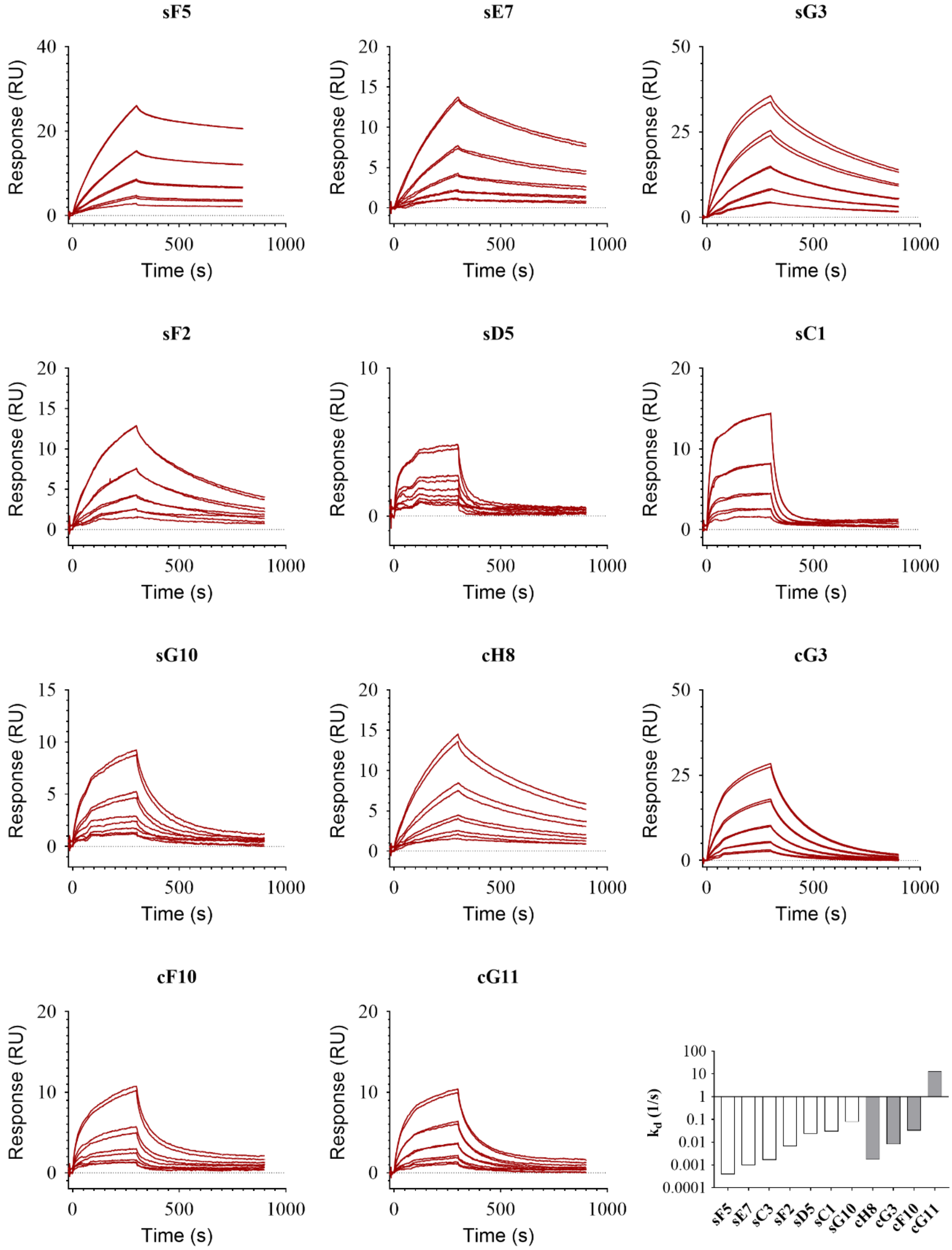

Supplemental Figure S11. Binding profile of anti-mβc mAbs by SPR. The recombinant ECD of mβc was captured on a sensor chip using covalently immobilized anti-His antibodies. Purified anti-mβc mAbs were used as analytes. Binding affinities (K_D) were not estimated because the avidity imposed by bivalent mAbs render these results inaccurate. Thus, antibody comparisons were limited to their apparent dissociation (k_d) rate constants. Dissociation values from these sensorgrams are shown in Table S4.

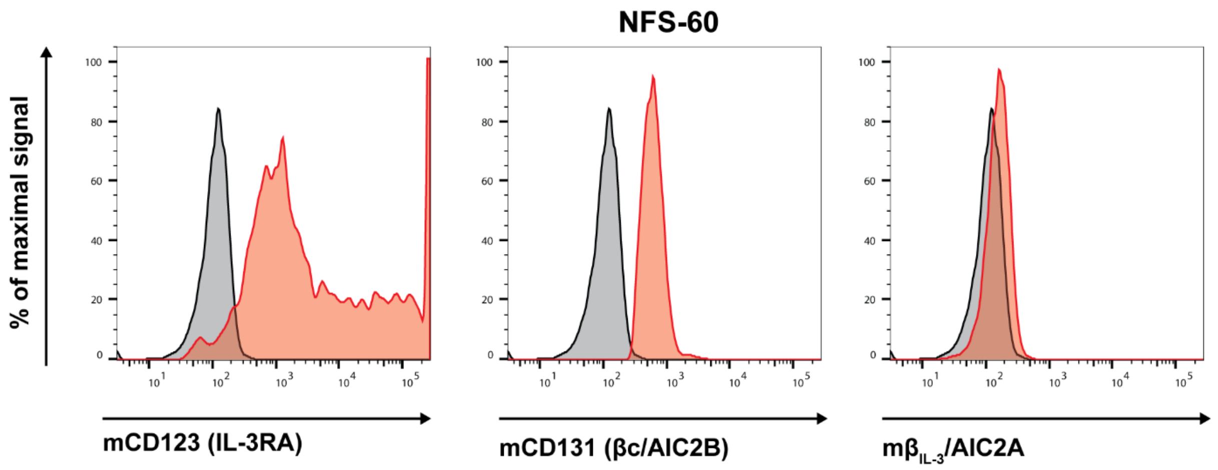

Supplemental Figure S12. Flow cytometry analysis of NFS-60 cells (red) with unstained control (grey). mIL-3Rα (left), mβc (middle) and $m\beta_{IL-3}$ (right) surface expression in NFS-60 cells.

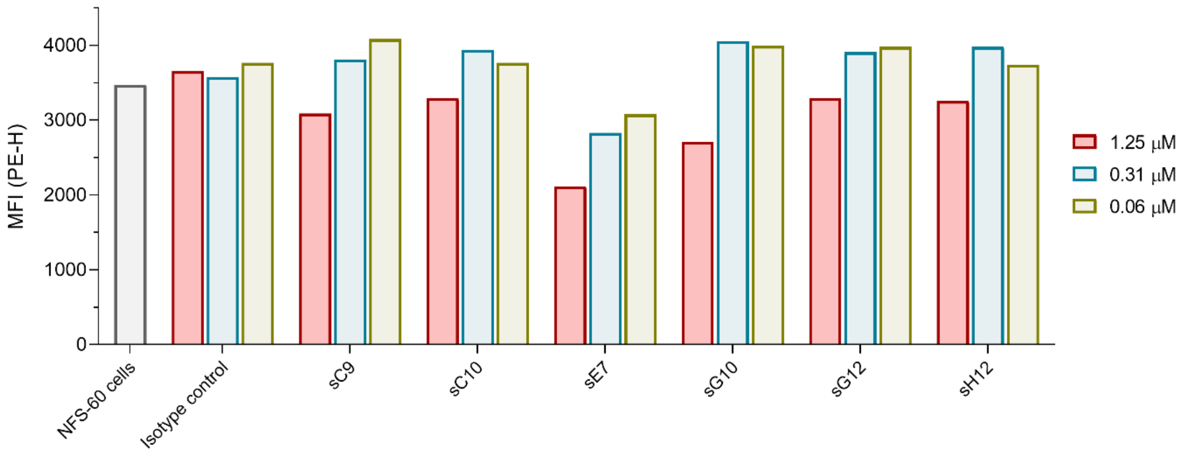

Supplemental Figure S13. Flow cytometric analysis of phosphorylated STAT5 (pSTAT5) levels in NFS-60 cells to determine the impact of anti-mβc mAbs on mIL-3 dependent signaling. Cells were supplemented with individual antibodies 30 min post addition of 2 ng/mL IL-3. pSTAT5 levels in permeabilized cells were probed with an PE conjugated mouse anti-pSTAT5 antibody.

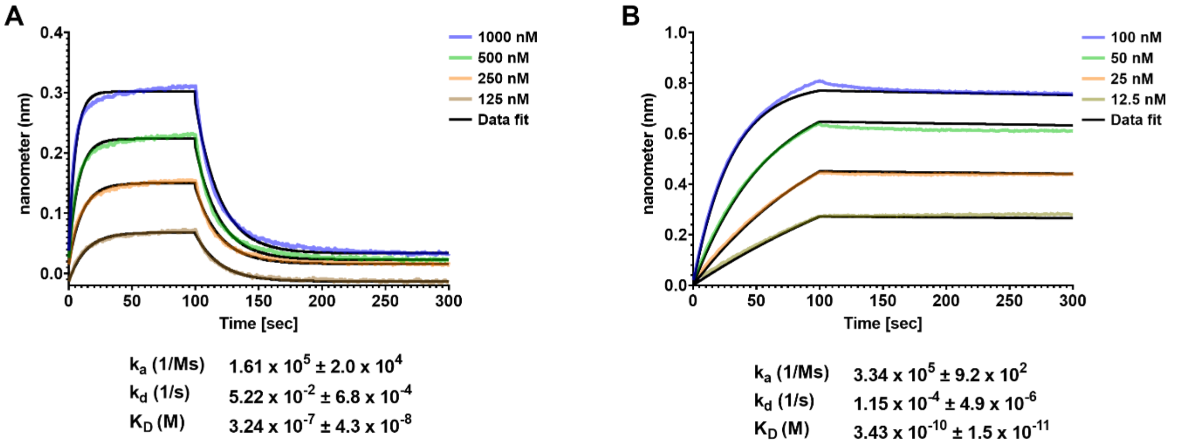

Supplemental Figure S14. Binding kinetics of antibody candidate sE7 assessed by biolayer interferometry (BLI). The recombinant mβc extracellular domain was immobilized to Ni-NTA biosensors and probed with (A) the scFv and (B) the full-length sE7 IgG molecule at varying concentrations. The overall equilibrium dissociation constant K_D of the full-length antibody is impacted by the avidity imposed by the bivalency of the molecule. Experimental sensorgrams (colored) were fitted with a 1:1 stoichiometric binding model (black).
